# Supplementary material for: SMOC-1 interacts with both BMP and glypican to regulate BMP signaling in C. elegans
Source: PLoS Biol. 2023 Aug 17;21(8):e3002272. doi: 10.1371/journal.pbio.3002272 (PMC10464977; doi:10.1371/journal.pbio.3002272)
Supplement: S1 Table — (PDF) [file pbio.3002272.s001.pdf]

Supplementary table S1: LON-2 peptides recovered from the IP-MS experiments.

1) IP using full length SMOC-1::2xFLAG (experiment 1)

[K].MAMTISEAIYR.[Y]
[R].NILTTENAISLTGIK.[Y]
[K].VISIDHDLLPITK.[D]
[K].SFLDEFLSLK.[K]
[R].KICDHSGVVLNPTMCYDGTK.[V]
[K].HWSVYLGNTPNK.[M]
[K].HSVHFQSPGPFVSR.[G]
[K].ICDHSGVVLNPTMCYDGTK.[V]
[K].ICDHSGVVLNPTMCYDGTK.[V]
[R].KVEFLLVDMHK.[Q]
[R].DAIAFTTGEK.[K]
[R].NILTTENAISLTGIK.[Y]
[K].GPSVIVTEK.[E]

2) IP using full length SMOC-1::2xFLAG (experiment 2)

[R].DAIAFTTGEK.[K]
[K].GPSVIVTEK.[E]
[K].HSVHFQSPGPFVSR.[G]
[K].HWSVYLGNTPNK.[M]
[R].KICDHSGVVLNPTMCYDGTK.[V]
[R].KVEFLLVDMHK.[Q]
[K].ICDHSGVVLNPTMCYDGTK.[V]
[K].ICDHSGVVLNPTMCYDGTK.[V]
[K].MAMTISEAIYR.[Y]
[K].NIDLSTNPK.[G]
[R].NILTTENAISLTGIK.[Y]
[R].NILTTENAISLTGIK.[Y]
[K].SFLDEFLSLK.[K]
[K].SYVYAEHVFNSCGPLGEMIIHPSK.[H]
[K].SYVYAEHVFNSCGPLGEMIIHPSK.[H]
[K].SYVYAEHVFNSCGPLGEMIIHPSK.[H]
[K].VISIDHDLLPITK.[D]
[K].YEVSTAVQK.[F]

3) IP using SMOC-1(EC)::2xFLAG

[R].DAIAFTTGEK.[K]
[K].GPSVIVTEK.[E]
[K].HSVHFQSPGPFVSR.[G]
[K].HWSVYLGNTPNK.[M]
[R].KICDHSGVVLNPTMCYDGTK.[V]
[R].KVEFLLVDMHK.[Q]
[K].ICDHSGVVLNPTMCYDGTK.[V]
[K].ICDHSGVVLNPTMCYDGTK.[V]
[K].MAMTISEAIYR.[Y]
[K].NIDLSTNPK.[G]
[R].NILTTENAISLTGIK.[Y]
[K].SFLDEFLSLK.[K]
[K].SYVYAEHVFNSCGPLGEMIIHPSK.[H]
[K].VISIDHDLLPITK.[D]
[K].YEVSTAVQK.[F]
