# Supplementary material for: SMOC-1 interacts with both BMP and glypican to regulate BMP signaling in C. elegans
Source: PLoS Biol. 2023 Aug 17;21(8):e3002272. doi: 10.1371/journal.pbio.3002272 (PMC10464977; doi:10.1371/journal.pbio.3002272)
Supplement: S4 Table — (PDF) [file pbio.3002272.s004.pdf]

Supplementary table S4. Plasmids generated in this study.

Plasmid ID	Details
**Plasmids for expression in *C. elegans***	
pMSD35	*smoc-1p::smoc-1 gDNA::2xflag::smoc-1 3'UTR*
pMSD43	*smoc-1p::smoc-1(D219N, D223N) gDNA::2xflag::smoc-1 3'UTR*
pMSD44	*smoc-1p::smoc-1 (TY) gDNA::2xflag::smoc-1 3'UTR*
pMSD45	*smoc-1p::smoc-1(EC) gDNA::2xflag::smoc-1 3'UTR*
pMSD46	*smoc-1p::smoc-1(jj65(C210Y)) gDNA::2xflag::smoc-1 3'UTR*
pMSD47	*smoc-1p::smoc-1(jj85(E105K)) gDNA::2xflag::smoc-1 3'UTR*
pMSD58	*smoc-1p::smoc-1 gDNA:: 2xflag::TM::smoc-1 3'UTR*
pMSD60	*smoc-1p::smoc-1(D219N, D223N, D229, E230N) gDNA::2xflag::smoc-1 3'UTR*
pMSD64	*smoc-1p::hsmoc1(EC) cDNA::2xflag::smoc-1 3'UTR*
pMSD65	*smoc-1p::hsmoc2(EC) cDNA::2xflag::smoc-1 3'UTR*
pMSD71	*smoc-1p::smoc-1(EC) gDNA:: 2xflag::TM::smoc-1 3'UTR*
pJKL1252	*smoc-1p::smoc-1(S152D S156D M160D) cDNA::V5::unc-54 3'UTR*
pJKL1253	*smoc-1p::smoc-1(F253D L257D) cDNA::V5::unc-54 3'UTR*
pJKL1254	*smoc-1p::smoc-1(Y72A Y90A Y95A W97A) cDNA::V5::unc-54 3'UTR*
pMSD1	sgRNA plasmid #1 for generating *smoc-1::2xflag* and *smoc-1(EC)::2xflag (in pRB1017)*<br>MSD-1: TCTTGATTCTGATCTTAAAATGTAC<br>MSD-2: AAACGTACATTTTAAGATCAGAATC
pMSD2	sgRNA plasmid #2 for *smoc-1::2xflag* and *smoc-1(EC)::2xflag (in pRB1017)*<br>MSD-3: TCTTGAACATTGCAAATTGAGGGGG<br>MSD-4: AAACCCCCCTCAATTTGCAATGTTC
pMSD67	sgRNA plasmid #1 for generating *smoc-1(TY)::2xflag (in pRB1017)*<br>MSD-163: TCTTGAGAAGAGAACACGATTTCTG<br>MSD-164: AAACCAGAAATCGTGTTCTCTTCTC
pMSD68	sgRNA plasmid #2 for generating *smoc-1(TY)::2xflag (in pRB1017)*<br>MSD-165: TCTTGGAGAAGAAACAATCGGTGTA<br>MSD-166: AAACTACACCGATTGTTTCTTCTCC
pMSD78	sgRNA plasmid #3 for generating *smoc-1(TY)::2xflag (in pRB1017)*<br>MSD-187: TCTTGGAAGGAGCATCGGGATCCAG<br>MSD-188: AAACCTGGATCCCGATGCTCCTTCC
pMSD79	sgRNA plasmid #4 for generating *smoc-1(TY)::2xflag (in pRB1017)*<br>MSD-189: TCTTGCTGTACATTTTAAGATCAGA<br>MSD-190: AAACTCTGATCTTAAAATGTACAGC
pMSD84	Repair template for generating *smoc-1(EC)::2xflag*
pJKL1204	sgRNA plasmid #1 for generating *HA::dbl-1 (in pRB1017)*<br>JKL-1840: TCTTGGTAGAAAGCATCATAACACCG<br>JKL-1841: AAACCGGTGTTATGATGCTTTCTAC
pJKL1205	sgRNA plasmid #2 for generating *HA::dbl-1 (in pRB1017)*

	JKL-1842: TCTTGCCTCCGACAAAGATTGCTCT<br>JKL-1843: AAACAGAGCAATCTTTGTCGGAGG
pTYC3	*dbl-1p::HA::dbl-1 gDNA::dbl-1 3'UTR*

**Plasmids for expression in *Drosophila* S2 cells**	
pJKL1210	*pAc5::sax-7ss::smoc-1::V5*
pJKL1211	*pAc5::sax-7ss::HA::smoc-1*
pMSD49	*pAc5::sax-7ss::smoc-1(TY)::V5*
pMSD50	*pAc5::sax-7ss::smoc-1(EC)::V5*
pMSD54	*pAc5::sax-7ss::HA::smoc-1(TY)*
pMSD55	*pAc5::sax-7ss::HA::smoc-1(EC)*
pJKL1249	*pAc5::sax-7ss::HA::smoc-1(S152D S156D M160D)*
pJKL1250	*pAc5::sax-7ss::HA::smoc-1(F253D L257D)*
pJKL1251	*pAc5::sax-7ss::HA::smoc-1(Y72A Y90A Y95A W97A)*
pCB313	*pAc5::sax-7ss::HA::lon-2::myc (gift from C. Benard)*
pJKL1241	*pAc5::sax-7ss::lon-2::myc*
pJKL1255	*pAc5::sax-7ss::lon-2 (S311D A315D F319D)::myc*
pJKL1198	*pAc5::HA::dbl-1pro::FLAG::dbl-1mature*
pJKL1199	*pAc5::V5::dbl-1pro::FLAG::dbl-1mature*

All plasmids were verified by Sanger sequencing.
