# Supplementary material for: SMOC-1 interacts with both BMP and glypican to regulate BMP signaling in C. elegans
Source: PLoS Biol. 2023 Aug 17;21(8):e3002272. doi: 10.1371/journal.pbio.3002272 (PMC10464977; doi:10.1371/journal.pbio.3002272)
Supplement: S5 Table — (PDF) [file pbio.3002272.s005.pdf]

Supplementary table S5. Oligonucleotides used in this study

Oligo ID	Sequence
***smoc-1::2xflag* repair oligo**	
MSD-67	CCAGCCAAACGTCCAGATCAACTAAACCCATTTCTGTACATTTTAAGATCAGAAGGAGCATCGGGATCCAGTGGA GCATCGGATTATAAAGACGATGACGATAAGCGTGACTACAAGGACGACGACGACAAGCGTTAATTTTAAGTTTTA ATTCTCCCCCTCAATTTGCAATGTTCTTTAAAAATCTACCA
***smoc-1(TY)::2xflag* repair oligo**	
MSD-191	GTGAAGAAACTCCAACAACTACAGTTGCTCCAAAAAGAGTGAGAAGAGGAGCTTCCGGTTCTAGTGGAGCATCG GATTATAAAGACGATGACG
crRNA guide sequences for making smoc-1(S152D S156D M160D)::2xflag	
JKL-2055	AAATCAGAAATGATAATGAG
JKL-2066	GAAGAGAACACGATTTCTG
***smoc-1(S152D S156D M160D)::2xflag* repair oligo**	
JKL--2057	CAATCGGTGTAAGGAAAAGAAGAGAACACGATTTCTGCGTCGATTAGTAGACACTCTCAAAGACGAGATGATCGA CTCCGGAATAAATGCAACAAAAGTTAGCAGAGATTCAGC
crRNA guide sequences for making smoc-1(F253D L257D)::2xflag	
JKL-2064	TTTAAGATCAGAAGGAGCAT
***smoc-1(F253D L257D)::2xflag* repair oligo**	
JKL--2065	GAGTACCAGCCAAACGTCCAGATCAACTAAACCCAgacCTcTACATcgaccGtTCAGAAggagcatcTggatccagtggagcatc gGATTATAAAGACGATGACG
crRNA guide sequences for making smoc-1(Y90A Y95A W97A)::2xflag	
JKL-2066	TTCAATTTATTGTTGGTGTG
***smoc-1(F253D L257D)::2xflag* repair oligo**	
JKL--2067	GTTAAGTTTACTCAATTTTCAGATTATATGATAAAGTGCAATGCGCTGATGTTTCAATTGCCTGCGCTTGCGTCGA CGAACTGTCCGGAGAGCCAAAGTTAGGCTCAT
***HA::dbl-1* repair oligo**	
MSD-110	TTGTAGGGTAGAAAGCATCATAACACCGAAGCTGAGGGATCCAGTGGAGCATCGTACCCATACGACGTCCCAGA CTACGCCGGAGCATCGGGATCCAGTAGCAATCTTTGTCGGAGGACTGATTTCTACGTGG
crRNA guide sequences for making lon-2(S311D A315D F319D)	
JKL-2060	ACGCAAGTCATATGTCTACG
***lon-2(S311D A315D F319D)* repair oligo**	
JKL--2061	CTTCTCTGTAATTCGAATTTTCATCGAACGCAAGGACTATGTCTACGACGAACATGTCGACAACTCGTGCGGTCC GCTTGGCGAGATGATCATTC
**For amplification of *smoc-1::2xflag* from *jj276***	
JKL-1549	ATCTAGCCCGGGTTTCCCCCATCTACAATCATCCAAGTTTTG
JKL-1550	TATCTCGGGCCCTTATTGCGAATGATAAACCCATTCAAGCTG
**For genotyping *jj276***	
MSD-10	AGAATGTCAGACAGTGCTCC
MSD-70	GACTACAAGGACGACGACGA

JKL-1211	TAATGGAAGGAGGTTACCCG
**For genotyping *jj411/412***	
MSD-10	AGAATGTCAGACAGTGCTCC
MSD-28	GCCAAAGTTAGGCTCATCGACAACAAGAGG
JKL-1211	TAATGGAAGGAGGTTACCCG
**For genotyping *jj441***	
JKL-1517	AGAAAGGGGGACTTGGTAGG
JKL-1519	GGTTTCCATTCACTTCTTTCAAGCAC
MSD-66	CGTATACATATTTGTTAAGTTTACTCAATTTTCAG
**For genotyping *jj499/500/501 [smoc-1(S152D S156D M160D)]***	
MSD-28	GCCAAAGTTAGGCTCATCGACAACAAGAGG
JKL-1519	GGTTTCCATTCACTTCTTTCAAGCAC
JKL-2058	AAGACGAGATGATCGACTCC
**For genotyping *510/511 [smoc-1(F253D L257D)]***	
MSD-10	AGAATGTCAGACAGTGCTCC
JKL-1211	TAATGGAAGGAGGTTACCCG
JKL-2068	CAGACCTCTACATCGACCGT
**For genotyping *smoc-1(Y90A Y95A W97A)***	
JKL-2069	GAAAAACTGACGGAGATGCTAG
JKL-1519	GGTTTCCATTCACTTCTTTCAAGCAC
JKL-2070	CAATTGCCTGCGCTTGCGTC
**For genotyping *ok4128***	
JKL-1981	CTGACAAGCCAGTTCACGAG
JKL-1982	CCCAGATTTCGGAACCTCAC
JKL-1983	CAACAAACCACCAACACATGAG
**For genotyping *jj307/8/9***	
ZL-499	AGACACAGAGCAGCTCACTGAGC
MSD-99	GATAAGCATCGTAGCCCTCTG
**For genotyping *jj507/508 [lon-2(S311D A315D F319D)]***	
JKL-1985	CCGACCCCTTTCCTCATGATT
JKL-2062	GTCTACGACGAACATGTCGA

JKL-2063	TCTCACGGCTCCGTCTCCTC
**For genotyping *e678***	
JKL-1053	TTGTATTGCTCTACCGGTCC
JKL-1054	TTGCCCGGAATTTCAACTGC
JKL-1055	TCAACTTACGGAAGCGATCG
**For genotyping *cc604***	
MLF-69	CGCAACAAGTTCATTCTCCA
MLF-70	CTTGGCTAAGATCCCATGCT
LW-40	TCCGACTTGACACTTCATCAGC
