# Supplementary material for: SMOC-1 interacts with both BMP and glypican to regulate BMP signaling in C. elegans
Source: PLoS Biol. 2023 Aug 17;21(8):e3002272. doi: 10.1371/journal.pbio.3002272 (PMC10464977; doi:10.1371/journal.pbio.3002272)
Supplement: S6 Table — (PDF) [file pbio.3002272.s006.pdf]

Supplementary table S6. *C. elegans* strains used in this study

Strain ID	Genotype
**Strains carrying the *smoc-1(tm7125)* null mutation**	
LW4834	*arIs37[secreted CC::gfp] I; smoc-1(tm7125) V; sma-9(cc604) X, isolate #1*
LW4441	*arIs37[secreted CC::gfp] I; smoc-1(tm7125) V; sma-9(cc604) X, isolate #2*
LW4442	*arIs37[secreted CC::gfp] I; smoc-1(tm7125) V; sma-9(cc604) X, isolate #3*
**Strains carrying endogenously tagged, non-functional, SMOC-1::GFP::3xFLAG**	
LW5428	*smoc-1(jj269[smoc-1::gfp::3xflag]) V*
LW5429	*smoc-1(jj271[smoc-1::gfp::3xflag]) V*
LW5469	*arIs37[secreted CC::gfp] I; smoc-1(jj269[smoc-1::gfp::3xflag]) V; sma-9(cc604) X*
**Strains carrying endogenously tagged SMOC-1::2xFLAG**	
LW5524	*smoc-1(jj276[smoc-1::2xflag]) V*
LW5525	*arIs37[secreted CC::gfp] I; smoc-1(jj276[smoc-1::2xflag]) V; sma-9(cc604) X, isolate #1*
LW5527	*arIs37[secreted CC::gfp] I; smoc-1(jj276[smoc-1::2xflag]) V; sma-9(cc604) X, isolate #2*
**Strains overexpressing untagged SMOC-1**	
LW4812	*ccIs4438[intrinsic CC::gfp] III; sma-9(ok1628) X; jjEx4812[pJKL1138(smoc-1p::smoc-1) + LiuFD188(myo-2p::mCherry)], line #1*
LW4813	*ccIs4438[intrinsic CC::gfp] III; sma-9(ok1628) X; jjEx4812[pJKL1138(smoc-1p::smoc-1) + LiuFD188(myo-2p::mCherry)], line #2*
LW5130	*jjIs5119[pMSD4.4(smoc-1p::smoc-1) + LiuFD188(myo-2p::mCherry)], x3*
**Strains overexpressing SMOC-1::2xFLAG**	
LW5798	*jjIs5798[pMSD35.7(smoc-1p::smoc-1::2xflag) + LiuFD290(ttx-3p::RFP)], x0*
LW5799	*jjIs5799[pMSD35.7(smoc-1p::smoc-1::2xflag) + LiuFD290(ttx-3p::RFP)], x0*
LW5800	*jjIs5800[pMSD35.7(smoc-1p::smoc-1::2xflag) + LiuFD290(ttx-3p::RFP)], x0*
LW5812	*jjIs5798[pMSD35.7(smoc-1p::smoc-1::2xflag) + LiuFD290(ttx-3p::RFP)], x2 isolate #1*
LW5813	*jjIs5798[pMSD35.7(smoc-1p::smoc-1::2xflag) + LiuFD290(ttx-3p::RFP)], x2 isolate #2*
LW5814	*jjIs5799[pMSD35.7(smoc-1p::smoc-1::2xflag) + LiuFD290(ttx-3p::RFP)], x2 isolate #1*
LW5815	*jjIs5799[pMSD35.7(smoc-1p::smoc-1::2xflag) + LiuFD290(ttx-3p::RFP)], x2 isolate #2*
LW5816	*jjIs5800[pMSD35.7(smoc-1p::smoc-1::2xflag) + LiuFD290(ttx-3p::RFP)], x2 isolate #1*
LW5817	*jjIs5800[pMSD35.7(smoc-1p::smoc-1::2xflag) + LiuFD290(ttx-3p::RFP)], x2 isolate #2*
LW6061	*jjEx6061[pMSD35.7(smoc-1::2xflag) + LiuFD290(ttx-3p::RFP)]*
LW6062	*jjEx6062[pMSD35.7(smoc-1::2xflag) + LiuFD290(ttx-3p::RFP)]*

LW6087	*jjEx6087[pMSD35.7(smoc-1::2xflag) + LiuFD290(ttx-3p::RFP)]; smoc-1(tm7125) V*
LW6088	*jjEx6088[pMSD35.7(smoc-1::2xflag) + LiuFD290(ttx-3p::RFP)]; smoc-1(tm7125) V*
LW6091	*jjEx6091[pMSD35.7(smoc-1::2xflag) + LiuFD290(ttx-3p::RFP)]; arIs37[secreted CC::gfp] I; cup-5(ar465) III; smoc-1(tm7125) V; sma-9(cc604) X*
LW6109	*jjEx6109[pMSD35.7(smoc-1::2xflag) + LiuFD290(ttx-3p::RFP)]; arIs37[secreted CC::gfp] I; cup-5(ar465) III; smoc-1(tm7125) V; sma-9(cc604) X*
LW6110	*jjEx6110[pMSD35.7(smoc-1::2xflag) + LiuFD290(ttx-3p::RFP)]; arIs37[secreted CC::gfp] I; cup-5(ar465) III; smoc-1(tm7125) V; sma-9(cc604) X*
**Strains overexpressing SMOC-1::V5**	
LW6671	*jjIs6671[pJKL1242(smoc-1::V5) + pJKL499(myo-2p::gfp)]*
LW6701	*jjIs6671[pJKL1242(smoc-1::V5) + pJKL499(myo-2p::gfp)], x2 isolate #1*
LW6704	*jjIs6671[pJKL1242(smoc-1::V5) + pJKL499(myo-2p::gfp)], x2 isolate #2*
**Strains carrying endogenously tagged SMOC-1 truncations**	
LW6276	*smoc-1(jj411[smoc-1(TY)::2xflag]) V*
LW6277	*smoc-1(jj412[smoc-1(TY)::2xflag]) V*
LW6427	*arIs37[secreted CC::gfp] I; cup-5(ar465) III; smoc-1(jj411[smoc-1(TY)::2xflag]) V; sma-9(cc604) X isolate #1*
LW6429	*arIs37[secreted CC::gfp] I; cup-5(ar465) III; smoc-1(jj411[smoc-1(TY)::2xflag]) V; sma-9(cc604) X isolate #2*
LW6395	*smoc-1(jj441[smoc-1(EC)::2xflag]) V*
LW6425	*arIs37[secreted CC::gfp] I; smoc-1(jj441[smoc-1(EC)::2xflag]) V; sma-9(cc604) X isolate #1*
LW6427	*arIs37[secreted CC::gfp] I; smoc-1(jj441[smoc-1(EC)::2xflag]) V; sma-9(cc604) X isolate #2*
LW6427	*arIs37[secreted CC::gfp] I; cup-5(ar465) III; smoc-1(jj441[smoc-1(EC)::2xflag]) V; sma-9(cc604) X isolate #3*
LW6426	*ccIs4438[intrinsic CC::gfp] III; smoc-1(jj441[smoc-1(EC)::2xflag]) V; sma-9(ok1628) X isolate #1*
LW6428	*ccIs4438[intrinsic CC::gfp] III; smoc-1(jj441[smoc-1(EC)::2xflag]) V; sma-9(ok1628) X isolate #2*
**Strains overexpressing SMOC-1 truncations**	
LW6053	*jjEx6053[pMSD44.4(smoc-1(TY)::2xflag) + LiuFD290(ttx-3p::RFP)]*
LW6054	*jjEx6054[pMSD44.4(smoc-1(TY)::2xflag) + LiuFD290(ttx-3p::RFP)]*
LW6051	*jjEx6051[pMSD44.4(smoc-1(TY)::2xflag) + LiuFD290(ttx-3p::RFP)]; smoc-1(tm7125) V*
LW6092	*jjEx6092[pMSD44.4(smoc-1(TY)::2xflag) + LiuFD290(ttx-3p::RFP)]; smoc-1(tm7125) V*
LW6089	*jjEx6089[pMSD44.4(smoc-1(TY)::2xflag) + LiuFD290(ttx-3p::RFP)]; arIs37[secreted CC::gfp] I; cup-5(ar465) III; smoc-1(tm7125) V; sma-9(cc604) X*
LW6090	*jjEx6090[pMSD44.4smoc-1(TY)::2xflag) + LiuFD290(ttx-3p::RFP)]; arIs37[secreted CC::gfp] I; cup-5(ar465) III; smoc-1(tm7125) V; sma-9(cc604) X*
LW6057	*jjEx6057[pMSD45.4(smoc-1(EC)::2xflag) + LiuFD290(ttx-3p::RFP)]*
LW6058	*jjEx6058[pMSD45.4(smoc-1(EC)::2xflag) + LiuFD290(ttx-3p::RFP)]*
LW6052	*jjEx6052[pMSD45.4(smoc-1(EC)::2xflag) + LiuFD290(ttx-3p::RFP)]; smoc-1(tm7125) V*

LW6093	*jjEx6093[pMSD45.4(smoc-1(EC)::2xflag) + LiuFD290(ttx-3p::RFP)]; smoc-1(tm7125) V*
LW6117	*jjEx6117[pMSD45.4(smoc-1(EC)::2xflag) + LiuFD290(ttx-3p::RFP)]; arIs37[secreted CC::gfp] I; cup-5(ar465) III; smoc-1(tm7125) V; sma-9(cc604) X*
LW6118	*jjEx6118[pMSD45.4(smoc-1(EC)::2xflag) + LiuFD290(ttx-3p::RFP)]; arIs37[secreted CC::gfp] I; cup-5(ar465) III; smoc-1(tm7125) V; sma-9(cc604) X*
**Strains overexpressing SMOC-1 with various point mutations**	
LW6059	*jjEx6059[(pMSD46.1(smoc-1(jj65 C210Y)::2xflag) + LiuFD290(ttx-3p::RFP)]*
LW6060	*jjEx6060[(pMSD46.1(smoc-1(jj65 C210Y)::2xflag) + LiuFD290(ttx-3p::RFP)]*
LW6077	*jjEx6077[pMSD47.7(smoc-1(jj85 E105K)::2xflag) + LiuFD290(ttx-3p::RFP)]*
LW6083	*jjEx6083[pMSD46.1(smoc-1(jj65 C210Y)::2xflag) + LiuFD290(ttx-3p::RFP)]; smoc-1(tm7125) V*
LW6084	*jjEx6084[pMSD46.1(smoc-1(jj65 C210Y)::2xflag) + LiuFD290(ttx-3p::RFP)]; smoc-1(tm7125) V*
LW6085	*jjEx6085[pMSD47.7(smoc-1(jj85 E105K)::2xflag) + LiuFD290(ttx-3p::RFP)]; smoc-1(tm7125) V*
LW6086	*jjEx6086[pMSD47.7(smoc-1(jj85 E105K)::2xflag) + LiuFD290(ttx-3p::RFP)]; smoc-1(tm7125) V*
**Strains carrying endogenously tagged SMOC-1(M1) with S152D S156D M160D mutations**	
LW6761	*smoc-1(jj499 jj276[smoc-1(S152D S156D M160D)::2xflag]) V*
LW6762	*smoc-1(jj500 jj276[smoc-1(S152D S156D M160D)::2xflag]) V*
LW6763	*smoc-1(jj501 jj276[smoc-1(S152D S156D M160D)::2xflag]) V*
LW6777	*smoc-1(jj499 jj276[smoc-1(S152D S156D M160D)::2xflag]) V; 2x outcrossed, isolate #1*
LW6778	*smoc-1(jj499 jj276[smoc-1(S152D S156D M160D)::2xflag]) V; 2x outcrossed, isolate #2*
LW6773	*smoc-1(jj500 jj276[smoc-1(S152D S156D M160D)::2xflag]) V; 2x outcrossed, isolate #1*
LW6774	*smoc-1(jj500 jj276[smoc-1(S152D S156D M160D)::2xflag]) V; 2x outcrossed, isolate #2*
**Strains carrying endogenously tagged SMOC-1(M1+M2) with S152D S156D M160D F253D L257D mutations**	
LW6787	*smoc-1(jj499 jj510 jj276[smoc-1(S152D S156D M160D F253D L257D)::2xflag]) V*
LW6788	*smoc-1(jj499 jj511 jj276[smoc-1(S152D S156D M160D F253D L257D)::2xflag]) V*
**Strains carrying endogenous LON-2(mut) with S311D A315D F319D mutations**	
LW6783	*lon-2(jj507[lon-2(S311D A315D F319D)] X*
LW6784	*lon-2(jj508[lon-2(S311D A315D F319D)] X*
**Strains carrying endogenously tagged HA::DBL-1**	
LW5863	*dbl-1(jj307[HA::dbl-1 active domain]) V*
LW5864	*dbl-1(jj308[HA::dbl-1 active domain]) V*
LW5865	*dbl-1(jj309[HA::dbl-1 active domain]) V*
LW5933	*dbl-1(jj308[HA::dbl-1]) V; ccIs4438[intrinsic CC::gfp] III; sma-9(cc604) X, isolate #1*

LW5934	*dbl-1(jj308[HA::dbl-1]) V; ccIs4438[intrinsic CC::gfp] III; sma-9(cc604) X, isolate #2*
**Mutant *dbl-1(0)* strain**	
LW4774	*Dbl-1(ok3749) V*
**Strains overexpressing tagged HA::DBL-1**	
LW6448	*jjIs6448[pTYC3(dbl-1p::HA::dbl-1 gDNA::dbl-1 3'UTR) + LiuFD188 (myo-2p::mCherry)] x0*
LW6530	*jjIs6448[pTYC3(dbl-1p::HA::dbl-1 gDNA::dbl-1 3'UTR) + LiuFD188 (myo-2p::mCherry)] x3*
**Strains overexpressing both SMOC-1::2xFLAG and HA::DBL-1**	
LW6592	*jjIs5799[pMSD35.7(smoc-1p::smoc-1::2xflag) + LiuFD290(ttx-3p::RFP)]; jjIs6448[pTYC3(dbl-1p::HA::dbl-1 gDNA::dbl-1 3'UTR) + LiuFD188 (myo-2p::mCherry)], isolate #1*
LW6593	*jjIs5799[pMSD35.7(smoc-1p::smoc-1::2xflag) + LiuFD290(ttx-3p::RFP)]; jjIs6448[pTYC3(dbl-1p::HA::dbl-1 gDNA::dbl-1 3'UTR) + LiuFD188 (myo-2p::mCherry)], isolate #2*
**Strains overexpressing SMOC-1::2xFLAG in the *lon-2(e678)* null background**	
LW6611	*jjIs5799[pMSD35.7(smoc-1p::smoc-1::2xflag) + LiuFD290(ttx-3p::RFP)]; lon-2(e678), isolate #1*
LW6612	*jjIs5799[pMSD35.7(smoc-1p::smoc-1::2xflag) + LiuFD290(ttx-3p::RFP)]; lon-2(e678), isolate #2*
**Strains overexpressing HA::DBL-1 in the *lon-2(e678)* null background**	
LW6614	*jjIs6448[pTYC3(dbl-1p::HA::dbl-1 gDNA::dbl-1 3'UTR) + LiuFD188 (myo-2p::mCherry)]; lon-2(e678), isolate #1*
LW6615	*jjIs6448[pTYC3(dbl-1p::HA::dbl-1 gDNA::dbl-1 3'UTR) + LiuFD188 (myo-2p::mCherry)]; lon-2(e678), isolate #2*
**Strain used for measuring body size of heterozygous hermaphrodite from a cross**	
LW3900	*jjIs3900(hlh-8p::NLS::mCherry::lacZ + myo-2p::mCherry) IV*
