# Supplementary figures and images for: SMOC-1 interacts with both BMP and glypican to regulate BMP signaling in C. elegans
Source: PLoS Biol. 2023 Aug 17;21(8):e3002272. doi: 10.1371/journal.pbio.3002272 (PMC10464977; doi:10.1371/journal.pbio.3002272)

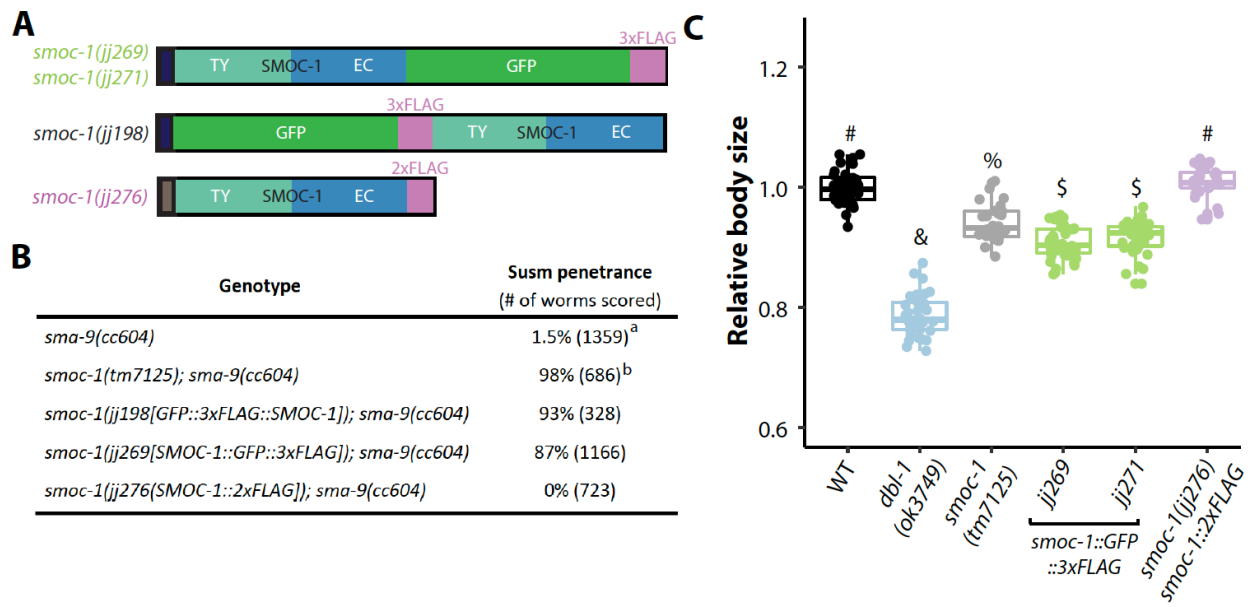

Supplement: S1 Fig — (A) Diagrams depicting various endogenously tagged SMOC-1 proteins, with the corresponding CRISPR alleles shown on the left of the diagrams. (B) Table showing the penetrance of the Susm phenotype of strains carrying specified smoc-1 allele in a sma-9(cc604) background. The Susm penetrance refers to the percent of animals with 1 or 2 M-derived CCs as scored using the arIs37(secreted CC::GFP) reporter. For each genotype, 2 independent isolates were generated (as shown in the strain list), 4 to 7 plates of worms from each isolate were scored for the Susm phenotype at 20°C, and the Susm data from the 2 isolates were combined and presented in the table. a The lack of M-derived CCs phenotype is not fully penetrant in sma-9(cc604) mutants [25]. b Data from [12]. Statistical analysis was conducted by comparing various double mutants with the sma-9(cc604) single mutant. **** P < 0.0001 (unpaired two-tailed Student’s t test). (C) Relative body sizes of various strains at the same developmental stage (WT set to 1.0). Body sizes of 35 to 70 worms of each genotype were measured. A dbl-1 null allele (ok3749) and smoc-1 null allele (tm7125) were included as controls. Groups marked with distinct symbols are significantly different from each other (P < 0.001, in all cases when there is a significant difference, except that the P-value between tm7125 and jj271 is 0.0099), while groups with the same symbol are not. The exception is tested using an ANOVA with a Tukey HSD. Original data sets are in S1 Data. (PDF) [file pbio.3002272.s007.pdf]

**A**

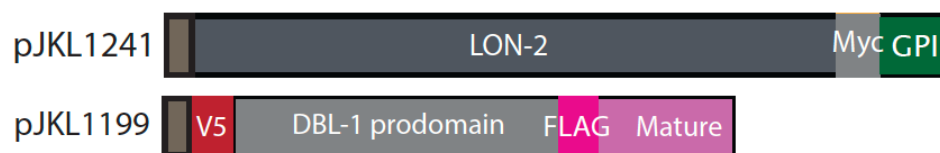

**B**

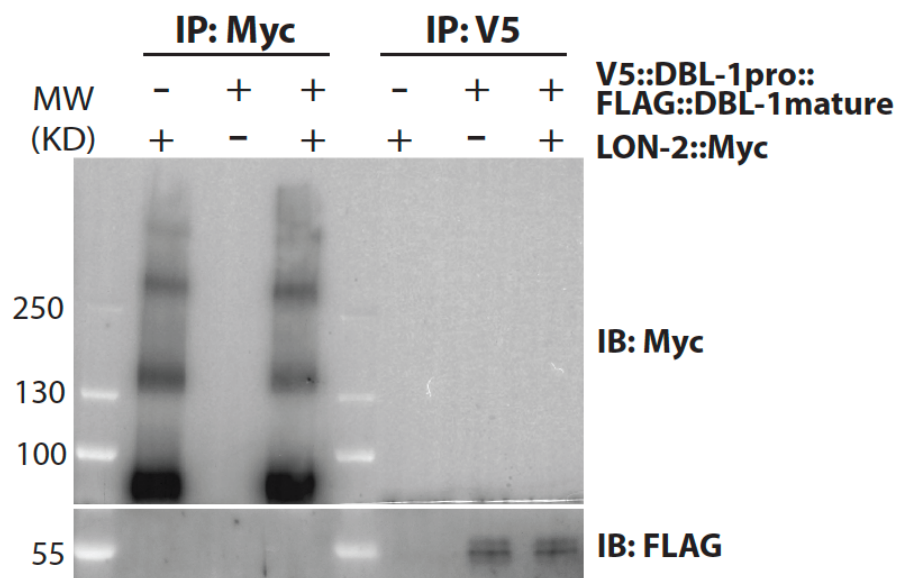

Supplement: S2 Fig — (A) Diagrams of LON-2 and DBL-1 expression constructs used in the Drosophila S2 cell expression system. (B) Results of co-IP experiments testing the interaction between LON-2::Myc and V5::DBL-1 prodomain::FLAG::DBL-1 mature domain. Immunoprecipitation (IP) with anti-Myc beads or anti-V5 beads and immunoblot (IB) with anti-Myc or anti-FLAG antibodies, as indicated. Full-length DBL-1 detected by anti-FLAG antibody runs at around 55KD. Experiments were independently repeated in triplicate, with representative results shown in this figure. Original images of western blots can be found in S1 Raw Images. (PDF) [file pbio.3002272.s008.pdf]

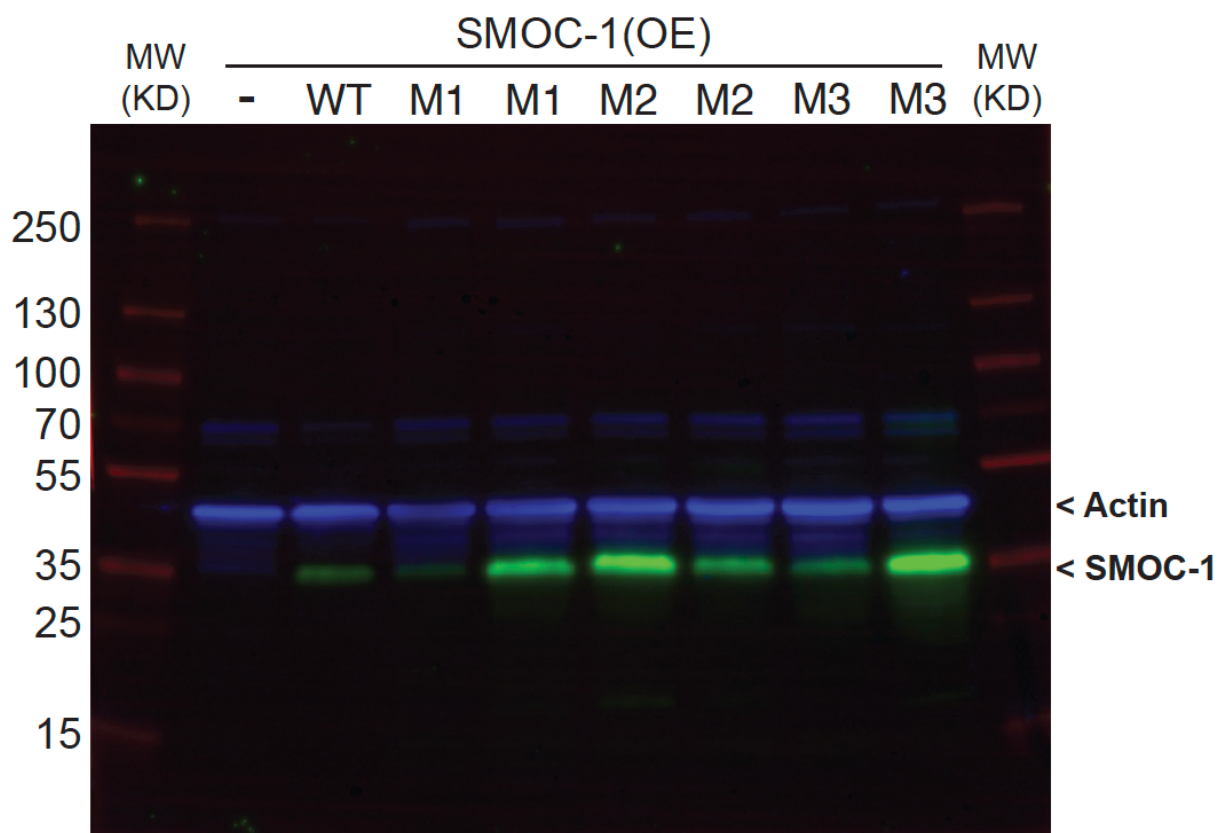

Supplement: S6 Fig — Western blot of 100 gravid adults of each indicated genotype, probed with anti-V5 (for SMOC-1) and anti-actin antibodies. The strain overexpressing WT SMOC-1::V5 is an integrated transgenic strain (jjIs6671). Strains overexpressing various mutant versions of SMOC-1::V5 (2 independent transgenic lines for each mutant version of SMOC-1) carry the transgenes as extra chromosomal arrays, thus the expression levels vary between different transgenic lines due to varying copy numbers of the transgene and varying degrees of stability of the transgenic arrays. (PDF) [file pbio.3002272.s012.pdf]

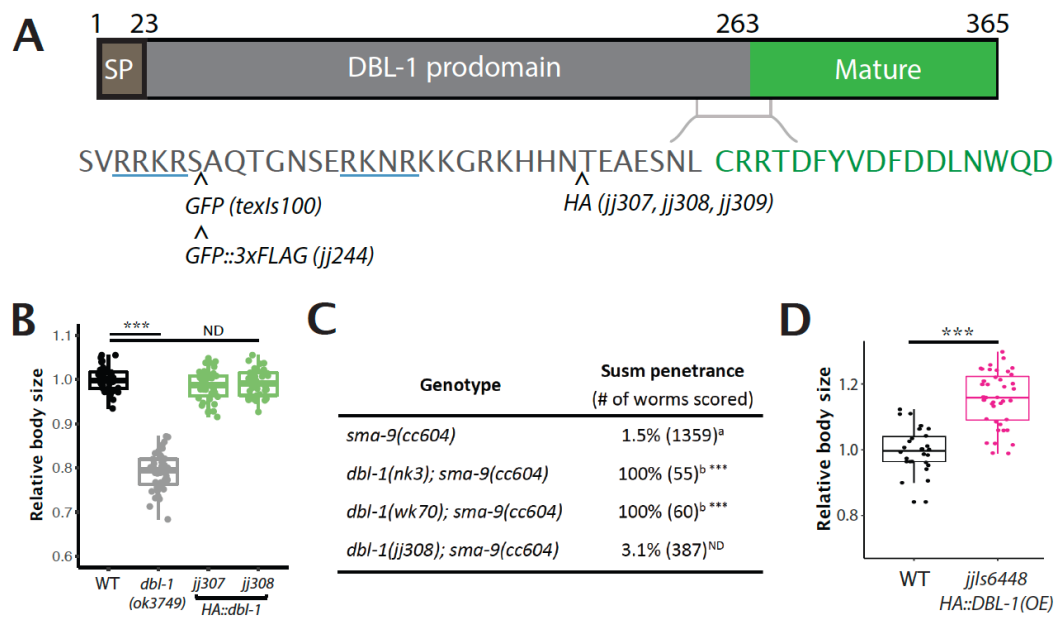

Supplement: S7 Fig — (A) Schematic of DBL-1 protein, which contains a signal peptide (SP, dark gray), a prodomain (light gray), and a cysteine-knot containing mature domain that is the active signaling ligand (green). Inset shows residues flanking the prodomain-mature domain boundary, indicating the predicted furin cleavage sites (underlined residues) in relation to the placements of the tags (arrows). texIs100 is an integrated transgene overexpressing a GFP-tagged DBL-1 [72]. However, when GFP was inserted in the same location as in texIs100 in the endogenous dbl-1 locus, the resulting allele, jj244, is not functional. jj307, jj308, and jj309 are 3 identical alleles generated via CRISPR, with the HA tag inserted in the marked location. (B) Relative body lengths of synchronized larvae stage-matched at L4.3 vulva stage grown at 20°C, with the body length of WT worms set to 1.0. Sample sizes are 40 for each genotype. An ANOVA followed by Tukey HSD was used to test for differences between genotypes. ***P < 0.001; ND, no difference. (C) Table showing the penetrance of the Susm phenotype of double mutant strains between sma-9(cc604) and different dbl-1 alleles. a The lack of M-derived CCs phenotype is not fully penetrant in sma-9(cc604) mutants. b Data for the 2 null dbl-1 alleles, nk3 and wk7, are from [25]. For jj308, 2 independent isolates were generated, and the Susm data from the 2 isolates were combined and presented in the table. Statistical analysis was conducted by comparing the strains carrying dbl-1 alleles with the sma-9(cc604) single mutants. *** P < 0.001 (unpaired two-tailed Student’s t test). ND, no difference. (D) Relative body lengths of stage-matched WT worms (set to 1.0) and worms carrying an integrated transgene (jjIs6448) that overexpresses HA::DBL-1. WT: N = 35. jjIs6446, N = 41. ***P < 0.001 (ANOVA followed by Tukey HSD). (E) Schematic of GFP::3xFLAG::LON-2 protein in lon-2(jj207) animals. Original data sets are in S1 Data. (PDF) [file pbio.3002272.s013.pdf]

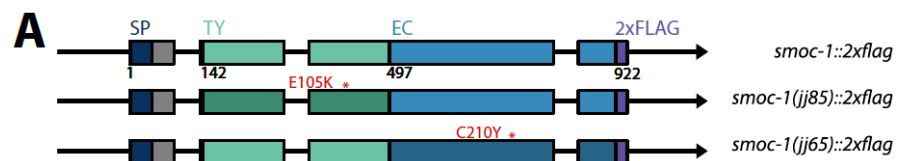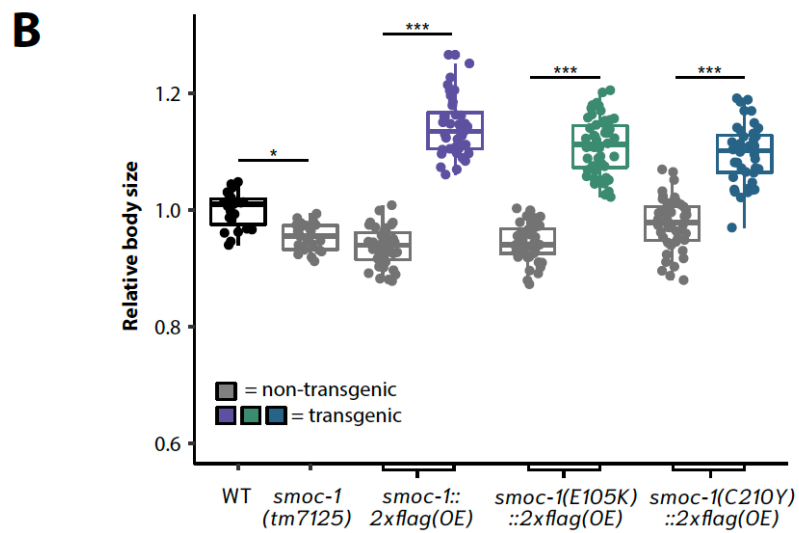

Supplement: S8 Fig — (A) Diagram depicting the constructs expressing WT smoc-1, smoc-1(jj85) (pMSD47), and smoc-1(jj65) (pMSD46). All plasmids contain the same 2 kb smoc-1 promoter and 2 kb smoc-1 3′ UTR, as well as a 2xFLAG tag at the C terminus. Protein domains are indicated by color: navy, SP, signal peptide; green, TY, thyroglobulin-like domain; blue, EC, extracellular calcium binding domain; purple, 2xFLAG. (B) Relative body sizes of strains carrying indicated versions of smoc-1 in a smoc-1(tm7125) null background relative to WT (set to 1.0). Gray indicates non-transgenic worms that do not express any smoc-1. Two strains carrying independent transgenes were measured and combined for each plasmid being tested here. Statistical analysis was done to compare transgenic strains with non-transgenic counterparts. *P < 0.01; ***P < 0.001; ND: no difference (ANOVA followed by Tukey HSD). Original data sets are in S1 Data. (PDF) [file pbio.3002272.s014.pdf]
