# Supplementary material for: SMOC-1 interacts with both BMP and glypican to regulate BMP signaling in C. elegans
Source: PLoS Biol. 2023 Aug 17;21(8):e3002272. doi: 10.1371/journal.pbio.3002272 (PMC10464977; doi:10.1371/journal.pbio.3002272)
Supplement: S3 Fig — Predicted alignment error (PAE) plots are shown for predictions involving BMP ligands produced by the ColabFold implementation of AlphaFold. PAE values are shown as a heat map, with blue representing low values and red representing high values. Blue represents residue pairs for which there is high confidence in their locations relative to each other in the 3D prediction. Red represents residue pairs with no confidence in their locations relative to each other in the 3D prediction. As indicated in the Key (top left), the regions of the plots within the dashed rectangles highlight the portions of the plot corresponding to predicted interactions between the BMP ligands and the protein of interest. The interaction between H. sapiens SMOC1 and the homodimeric BMP2 ligand is predicted with high confidence, as indicated by the overall low PAE values for the interacting residue pairs (top right). In contrast, the plots for H. sapiens glypican 1 with different combinations of BMP2/4 dimeric ligands, and for C. elegans LON-2 with the homodimeric DBL-1 ligand, show high predicted errors indicating a lack of predicted interaction. Similar negative results were observed for predictions involving full-length or prodomain sequences of the BMP ligands. (PDF) [file pbio.3002272.s009.pdf]

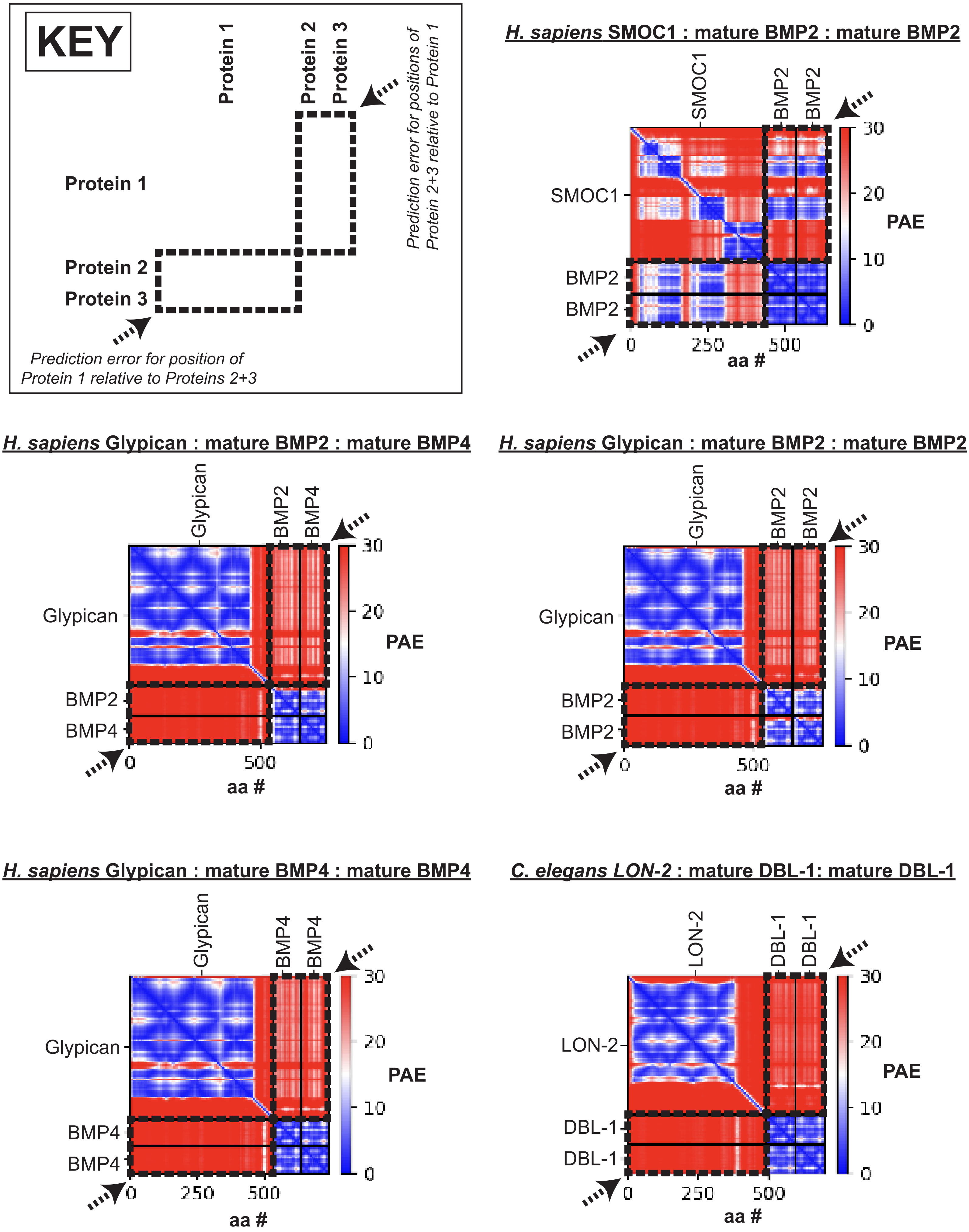
KEY
Protein 1
Protein 2
Protein 3
Prediction error for positions of Protein 2+3 relative to Protein 1
Prediction error for position of Protein 1 relative to Proteins 2+3
H. sapiens SMOC1 : mature BMP2 : mature BMP2
SMOC1
BMP2
BMP2
PAE
aa #
H. sapiens Glypican : mature BMP2 : mature BMP4
Glypican
BMP2
BMP4
PAE
aa #
H. sapiens Glypican : mature BMP2 : mature BMP2
Glypican
BMP2
BMP2
PAE
aa #
H. sapiens Glypican : mature BMP4 : mature BMP4
Glypican
BMP4
BMP4
PAE
aa #
C. elegans LON-2 : mature DBL-1: mature DBL-1
LON-2
DBL-1
DBL-1
PAE
aa #
