# Supplementary material for: SMOC-1 interacts with both BMP and glypican to regulate BMP signaling in C. elegans
Source: PLoS Biol. 2023 Aug 17;21(8):e3002272. doi: 10.1371/journal.pbio.3002272 (PMC10464977; doi:10.1371/journal.pbio.3002272)
Supplement: S4 Fig — Clustal Omega (CLUSTAL O(1.2.4)) [71] of C. elegans (Ce) LON-2 with its homologs from other nematode species, including C. Japonica (Cjp), C. brenneri (Cbn), C. briggsae (Cbr), and C. remanei (Cre), as well as with Drosophila Dally and Glypican from M. musculus (mouse) and H. sapiens (human). Red $ marks the residues at the interface between LON-2::SMOC-1, as identified via ColabFold [34] and #### marks the glycosaminoglycan attachment site. Residues highlighted in yellow are those mutated to generate LON-2(mut) that cannot bind SMOC-1. (PDF) [file pbio.3002272.s010.pdf]

```
Ce-LON-2            -MVFR---------WLILFVLLYRSVLP----A--------------------------- 19
Cjp-LON-2           MHFFR---------WLILLVLLRSSTPT-EDAE--------------------------- 23
Cbn-LON-2           -MNFR---------WLILLVFYFRSISP----T--------------------------- 19
Cbr-LON-2           -MNFR---------WLILLVFLFRTALP---LS--------------------------- 20
Cre-LON-2           -MNFR---------WLILLVLLFRTALS----S--------------------------- 19
Drosophila-Dally    -MAARSVRLAQLLLFTLLCGFVGLSAAKHLDLDGIHHHQHHLHSATTHHRRRLQRDSRAK 59
Mouse-Glypican      -MELR------TRGWWLLCAAAALVVCA-------------------------------R 22
Human-Glypican      -MELR------ARGWWLLCAAAALVACA-------------------------------R 22
                        *         : :*


Ce-LON-2            ----------EEV-VVV---DIL-----TNS---TSLEEPTEEYTC-DCNTDD-LIQKGN 55
Cjp-LON-2           ----------NEK-VVI---DLL-----TNT---TTVEE-SEDVIC-DCNGSD-MLEKAN 58
Cbn-LON-2           ----------DDA-VVF---DIL-----TNS---TSLEEPTEEYTC-ECDVEE-LTEKGN 55
Cbr-LON-2           ----------EEV-VIV---DTL-----TNS---TALEEPTDEYTC-DCNDKD-LLEKGN 56
Cre-LON-2           ----------EEV-VVV---DIL-----TNS---TSLEEPTDEFTC-ECDAED-LLEKGN 55
Drosophila-Dally    DAVGGSTHQCDAVKSYFESIDIKSSG--TYSEKGRHLRRKL--LQQCHGAGA---AGQSR 112
Mouse-Glypican      GDPASKSRSCSEVRQIYGAKGFSLSDVPQAEISGEHLRICPQGYTCCTSEMEENLANHSR 82
Human-Glypican      GDPASKSRSCGEVRQIYGAKGFSLSDVPQAEISGEHLRICPQGYTCCTSEMEENLANRSH 82
                                        .                :.                   :..

                                                          $$
Ce-LON-2            YTLTVEVKNMQVRLVEPFRDAIAFTTGEKKHLLDFIRFVTLREVRSTYPLLLNYTDFLNS 115
Cjp-LON-2           YTLTVEVKNMQVRLVEPFRDAIAFTTSEKQHLLRFVKYVTLRDIRSTYPMLLNYTDFYAN 118
Cbn-LON-2           YTLTIEVRNMQVRLVEPFRDAIAFVASEKQHLLDFIKFVTLRDIKADYPLLLNYTEFYDS 115
Cbr-LON-2           YTLTVEVKNMQVRLVEPFRDAIAFITSEKQHLLDYIQFVVLRDTKATYPLLLNYTEYASS 116
Cre-LON-2           YTLTVELRNMQVRLVEPFRDAIAFVASEKTHLLDFIKFVTLRDIRSTYPLLLNYTEFYTN  115
Drosophila-Dally    RNVRATA--SSP-HQQPARSPRDECQSHVLELAQISE----NMTHSLFSK--VYTRMVPS 163
Mouse-Glypican      MELESALHDSSRALQATLATQLHGIDDHFQRLLNDSE----RTLQEAFPG--AFGDLYTQ 136
Human-Glypican      AELETALRDSSRVLQAMLATQLRSFDDHFQHLLNDSE----RTLQATFPG--AFGELYTQ 136
                      :       .               ..  .*     .    .  :  :     :     .

                                                                        $  $
Ce-LON-2            ----FDEMIGTFRNILT-------TE---NAISLTGIKYEVSTAVQKFLSAILPDMFLCL 161
Cjp-LON-2           ----YDELLGTIANILL-------TE---NDISFSGIRYEVTTAVDKFLKSLLPDMFLCL 164
Cbn-LON-2           ----FDGLIGSLRGILT-------TD---NAISLSGIKYEVSTAVQKFLNKLLPDMFLCL 161
Cbr-LON-2           ----FDELIKTFKNILS-------TE---NDILLSGIKYEVTTAVQKFFTSLLPDMFLCL 162
Cre-LON-2           ----FDDLIGTFRNILT-------TE---NAISLSGIKYEVTTAVQKFLSALLPDMFLCL 161
Drosophila-Dally    SRMMIHQLYTEIMNHLIYTSNYTNSNGQLGRRGIGSVQSNLEEAVRHFFVQLFPVAYHQM 223
Mouse-Glypican      NTRAFRDLYAELR---------------LY---YRGANLHLEETLAEFWARLLERLFKQL 178
Human-Glypican      NARAFRDLYSELR---------------LY---YRGANLHLEETLAEFWARLLERLFKQL 178
                          :   :                        . . .:  :: .*    ::   :  :

$     $        $
Ce-LON-2            S---VGKCRTVPLDYHNCMMASTKHWSVYLGNTPNKMAMTISEAIYRYRKVEFLLV---D 215
Cjp-LON-2           S---VGKCRTVPLEYHNCMLENTKHWSTYIGNTPNKMAATISDTIYRYRKVEFLLV---D 218
Cbn-LON-2           S---VGKCRTVPSDYYNCMMASTEHWSVYLGNTPNKMAMTVSEAIYRYRKVEFLLV---D 215
Cbr-LON-2           S---VGKCRTVPLEYQNCMTASTEHWSVYIGNTPNKMAVTIAEAIYRYRKVEFLLV---D 216
Cre-LON-2           S---VGKCRTVPLEYHNCMMTSTEHWSEYIGNTPNKMAMTIADAIYRYRKVEFVLV---D 215
Drosophila-Dally    VHLSKNNLGDLHEDYVNCLQHNFDEMHP-FGDIPQQVQSNLGKSVHMSNVFMNALLQAAE 282
Mouse-Glypican      H-----PQL-LPDDYLDCLGKQAEALRP-FGDAPRELRLRATRAFVAARSFVQGLGVASD 231
Human-Glypican      H-----PQLLLPDDYLDCLGKQAEALRP-FGEAPRELRLRATRAFVAARSFVQGLGVASD 232
                              :  :* :*:  . .     :*: *.::      :.   . .   *    :


Ce-LON-2            MHKQLMNAHNLTLSHECLQEYVHTLPCN-C------TMAGITPCHTSCSNSMEKCFGKFS 268
Cjp-LON-2           MHKQLMNAHNLTLTDACLQEYVTTLPCN-C------TTSGIGRCKTSCSDAMEKCFGKYS 271
Cbn-LON-2           MHRQLMQAHSLTITDECLSEYVNTLPCN-C------TMLGIARCGTSCPNSMEKCFGKFS 268
Cbr-LON-2           MHQQLMNAQSLTITDECLKQYVHSLPCN-C------TLSGIVRCQTSCATSMETCFGKYS 269
Cre-LON-2           MHKQLMNAHSLTVTDGCLTEYVTTLPCN-C------TMLGIVRCHTSCSDSMETCFGKYS 268
Drosophila-Dally    VLSEADALYGEQLTDTCKLHLLKMHYCPNCNGHHSSSRSETKLCYGYCKNVMRGCSAEYA 342
Mouse-Glypican      VVRK---VAQVPLAPECSRAIMKLVYCAHCRGVPG-----ARPCPDYCRNVLKGCLANQA 283
Human-Glypican      VVRK---VAQVPLGPECSRAVMKLVYCAHCLGVPG-----ARPCPDYCRNVLKGCLANQA 284
                    :  :        :   *    :    *  *             *   *   :. * .: :
```

```
                                                                             $$  $$      $
Ce-LON-2              ----REWAAKLHLMRNMTSTKKSFLDEFLSLK---KTIFSVIRIFIERKSYVYAEHVFNS 321
Cjp-LON-2             ----RDWSAKLHLMRNMTNTKKRFLEGFLTLK---KTIFSAIRLFIERKSFVYAEHVFNS 324
Cbn-LON-2             ----REWAAKLHLMRNMTNSKKSYLEEFLTLK---KTIFQVIRQFIERKSFVYAEHVFNS 321
Cbr-LON-2             ----REWAAKLHLMRNMTSTKKSFLDEFLTLR---KTIFSVIRLFIERKSFEYAAYVYKA 322
Cre-LON-2             ----REWAAKLHLMKNMTSTKKSFLEEFLTLK---KTIFSVIRLFIERKSFLYAEQVFKS 321
Drosophila-Dally      GLLDSPWSGVVDSLNNLVTTHILSDTGIINVIKHLQTYFSEAIMAAMHNGPELEKKVKKT 402
Mouse-Glypican        D-LDAEWRNLLDSMV-LITDKFWGPSGAESVIGGVHVWLAEAINALQDNKDTLTAKVIQA 341
Human-Glypican        D-LDAEWRNLLDSMV-LITDKFWGTSGVESVIGSVHTWLAEAINALQDNRDTLTAKVIQG 342
                            *   :. :  :  . :        .:    :. :         :       * :

                         $
Ce-LON-2              CGPLGEMIIH-------PSKHS-----VHFQSPGPFVSRGDGAVRELQLSAKTWDRLGRK 369
Cjp-LON-2             CGPLGEMIIH-------PSKHI-----FHLFSPGPFVSRADGAVRELQLSTKMWEKLGRK 372
Cbn-LON-2             CGPLGQMIIH-------PSKHV-----TRFHSPGPFVSRADGAVKELQLSAKSWDRFGRK 369
Cbr-LON-2             CGPLGEMIIH-------PSKHV-----ARIHSPGPFVSRADGAVRELQLSAKSWDRFGRK 370
Cre-LON-2             CGPLGEMIIH-------PSKHV-----AQIHSPGPFVSRADNAVRELQLSAKSWDRFGRK 369
Drosophila-Dally      CGTPSLTPYSSGEPDARPPPHKNNVKWATDPDPGMVLF-----LSTIDKSKEFYTTIVDN 457
Mouse-Glypican        CGNPKVNPHGSGPEEKRRRG---KLALQEKPSTGTLEKLVSEAKAQLRDIQDFWISLPGT 398
Human-Glypican        CGNPKVNPQGPGPEEKRRRG---KLAPRERPPSGTLEKLVSEAKAQLRDVQDFWISLPGT 399
                      **                               * .          :    . :  :  .

Ce-LON-2              ICDHSGV--VLNPTMCYDGTKVISIDHDLL-PITKDVRPRPMMDWIEKK----------- 415
Cjp-LON-2             ICEHNGI--VYHPTLCFDGTKVISIEHELP-PITKDVRPRPMMDWIEKR----------- 418
Cbn-LON-2             ICDH-GV--VLHPGMCYDGTKVIT------------------------------------ 390
Cbr-LON-2             ICDHTGV--VVHPGMCYDGTKVIKVEHELL-PITKDVRPRPMMDWIEKK----------- 416
Cre-LON-2             ICDHTGV--VLHPGYCFDGTKVIKIQHELA-PITKDVRPKTMMDWIEKK----------- 415
Drosophila-Dally      FCDEQQH--SRDDHSCWSGDRFGDYTQLLINPGTDSQRYNPEVPFNAKAQTGKLNELVDK 515
Mouse-Glypican        LCSEKMAMSPASDDRCWNGISKGRYLPEVMGDGLANQINNPEVEVDITKPDMTIRQQIMQ 458
Human-Glypican        LCSEKMALSTASDDRCWNGMARGRYLPEVMGDGLANQINNPEVEVDITKPDMTIRQQIMQ 459
                      :*..           *:.*

                                                     ####
Ce-LON-2              ---NEKKA--NVEGSASNPLWDDDEDSEDFDGSGSGMPPV-IDRNPVKAIIQEDHPKNIDL 469
Cjp-LON-2             ---GEKKPTAVVEGS-ASPLWDDDEDSDDFEGSGSGLPTTIDNKEPTKT---EEPTKTIDL 471
Cbn-LON-2             ------------------------------------------------------------ 390
Cbr-LON-2             ---NEKKI--AVEGSA-SPLWDDDEDSDGFEGSGSGMPPIIIDRNPVKAVIQEDHPKNIDL 470
Cre-LON-2             ---NEKKI--AVEGSA-SPLWDDEDFDGFEGSGSGMPPNIIDRNPVKAVIQQDHPKNIDL 469
Drosophila-Dally      LFKIRKSIG-AAAPSNSIQATHDIQNDMGEGSGGGEGQIGDDEEEYGGAHGSG-DGSGDG 573
Mouse-Glypican        LKIMTNRLR-GAYGGNDVDFQD--ASDDGSGSGSGGGCPDDTC---GRRVSKK-SS---S 508
Human-Glypican        LKIMTNRLR-SAYNGNDVDFQD--ASDDGSGSGSGDGCLDDLC---GRKVSRK-SS---S 509

Ce-LON-2              STNPKGPS------VIVTEKEIQPDGSTTSSILICVII-VAVIKLF--------       508
Cjp-LON-2             SINPKGPS------VIANEQENSPEPDGSSTICPITIL-ALVLAFWCI------       512
Cbn-LON-2             ------------------------------------------------------       390
Cbr-LON-2             STNPKGPS------VIVTEQGSQPDGSSTVSALTSLII-IAIIRFF--------       509
Cre-LON-2             STNPKGPS------VIVTEQGVQPDGSSILTIFTSLIF-IAIIRLF--------       508
Drosophila-Dally      PHTPIEESEGTTTNEVESRDSGKTSGSNPLEGTATWMLLTLVTMLFSSCS----       623
Mouse-Glypican        SRTPLTHALP----GL-SEQEGQKTSAATCPEPHSFFLLFLVTLVLAAARPRWR       557
Human-Glypican        SRTPLTHALP----GL-SEQEGQKTSAASCPQPPTFLLPLLLFLALTVARPRWR       558
```
