# Supplementary material for: SMOC-1 interacts with both BMP and glypican to regulate BMP signaling in C. elegans
Source: PLoS Biol. 2023 Aug 17;21(8):e3002272. doi: 10.1371/journal.pbio.3002272 (PMC10464977; doi:10.1371/journal.pbio.3002272)
Supplement: S5 Fig — Clustal Omega (CLUSTAL O(1.2.4)) alignment [71] of the mature domains of DBL-1 with its homologs from other nematode species, as well as homologs from Drosophila, Xenopus, and humans. Red $ marks the residues at the interface between mature DBL-1 and SMOC-1, as identified by ColabFold [34]. The first cysteine residue in the mature domain of DBL-1 homologs is assigned as the #1 position. (PDF) [file pbio.3002272.s011.pdf]

```
                                 $    $ $$$$$ $
C.elegans_DBL-1         CRRTDFYVDFDDLNWQDWIMAPKGYDAYQCQGSCPNPMPAQLNATNHAIIQSLLHSLRPD  60
C.brenneri_DBL-1        CRRTDLYVDFDDLGWQDWIMAPKGYDAYQCQGSCPNPMPAQLNATNHAIIQSLLHSIKPD  60
C.remanei_DBL-1         CRRTDLYVDFDDLGWQDWIMAPKGYDAYQCQGSCPNPMPAHLNATNHAIIQSLLHSLKPD  60
C.briggsae_DBL-1        CRRTDLYVDFDDLGWQDWIMAPKGYDAYQCQGSCPNPMPAQLNATNHAIIQSLLHSLKPD  60
D.melanogaster_DPP      CRRHSLYVDFSDVGWDDWIVAPLGYDAYYCHGKCPFPLADHFNSTNHAVVQTLVNNMNPG  60
H.sapiens_BMP2          VGDPPLLVNFSDVGWNDWIVAPPGYHAFYCHGECPFPLADHLNSTNHAIVQTLVNSVN-S  59
X.laevis_BMP4           CRRHSLYVDFSDVGWNDWIVAPPGYQAFYCHGDCPFPLADHLNSTNHAIVQTLVNSVN-S  59
H.sapiens_BMP4          CRRHSLYVDFSDVGWNDWIVAPPGYQAFYCHGDCPFPLADHLNSTNHAIVQTLVNSVN-S  59
X.laevis_BMP2           CRRHPLYVDFSDVGWNDWIVAPPGYHAFYCHGECPFPLADHLNSTNHAIVQTLVNNVN-P  59
                             : *:*.*:.*:***:** **.*: *:*.** *:  ::*:****::*:*::.:.

                                        $$$$    $$$$$$$$ $
C.elegans_DBL-1         EVPPPCCVPTETSPLSILYMDVDKVIVIREYADMRVESCGCR  102
C.brenneri_DBL-1        EVPPPCCVPTETSPLSILYMDVDKVIVIREYADMRVDSCGCR  102
C.remanei_DBL-1         EVPPPCCVPTETSPLSILYMDVDKVIVIREYADMRVDSCGCR  102
C.briggsae_DBL-1        EVPPPCCVPTETSPLSILYMDVDKVIVIREYADMRVDSCGCR  102
D.melanogaster_DPP      KVPKACCVPTQLDSVAMLYLNDQSTVVLKNYQEMTVVGCGCR  102
H.sapiens_BMP2          KIPKACCVPTELSAISMLYLDENEKVVLKNYQDMVVEGCGCR  101
X.laevis_BMP4           SIPKACCVPTELSAISMLYLDEYDKVVLKNYQEMVVEGCGCR  101
H.sapiens_BMP4          SIPKACCVPTELSAISMLYLDEYDKVVLKNYQEMVVEGCGCR  101
X.laevis_BMP2           NIPKACCVPTELSAISMLYLDENEKVVLKNYQDMVVEGCGCR  101
                        .:*  *****: . :::**:: . :*:::* :* * .****
```
