# Supplementary material for: SMOC-1 interacts with both BMP and glypican to regulate BMP signaling in C. elegans
Source: PLoS Biol. 2023 Aug 17;21(8):e3002272. doi: 10.1371/journal.pbio.3002272 (PMC10464977; doi:10.1371/journal.pbio.3002272)

Raw image files for Figure 1B

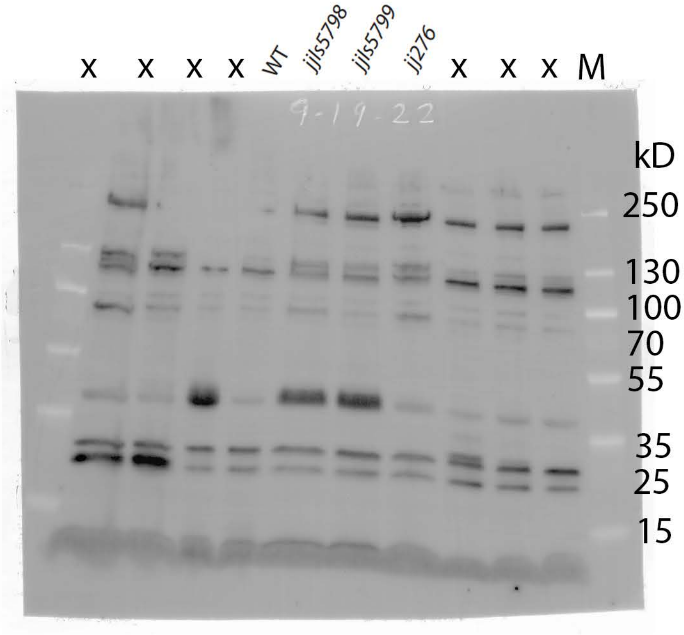

Anti-FLAG

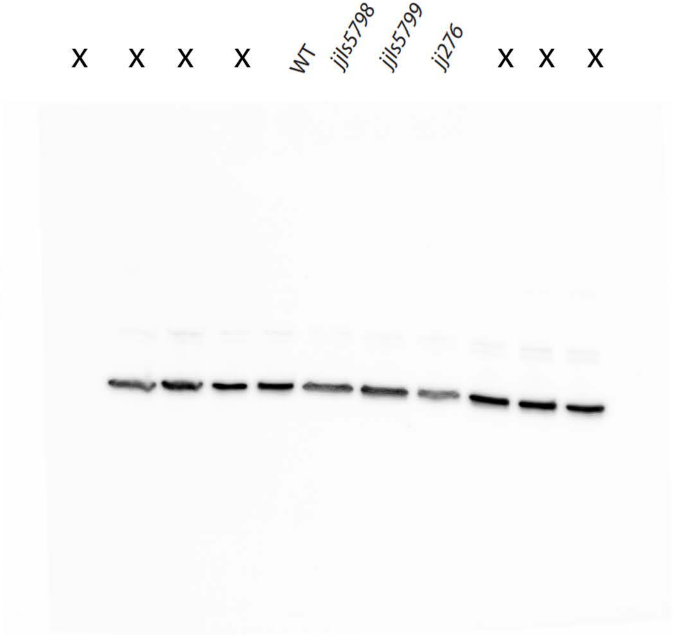

Anti-Actin

Raw image files for Figure 2C

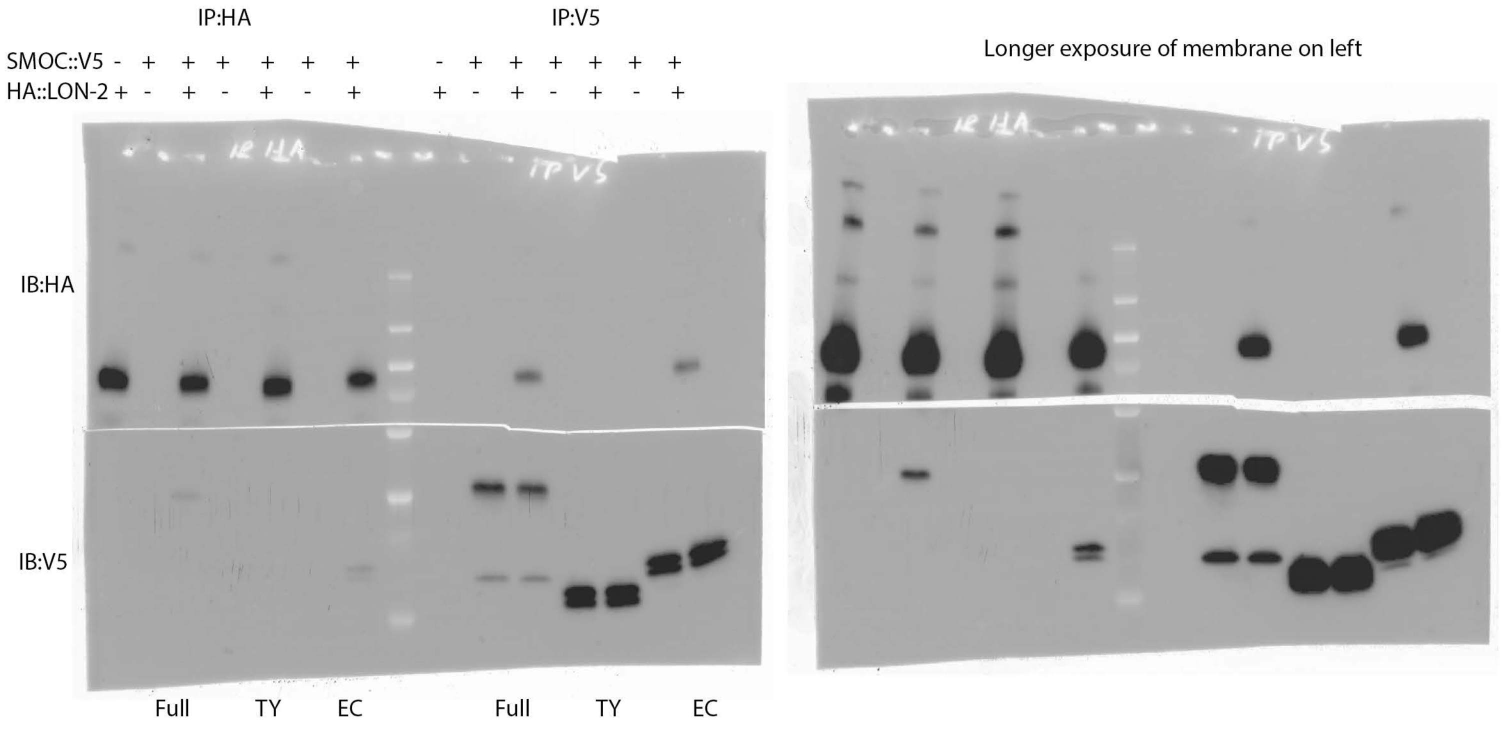

Raw image files for Figure 3B

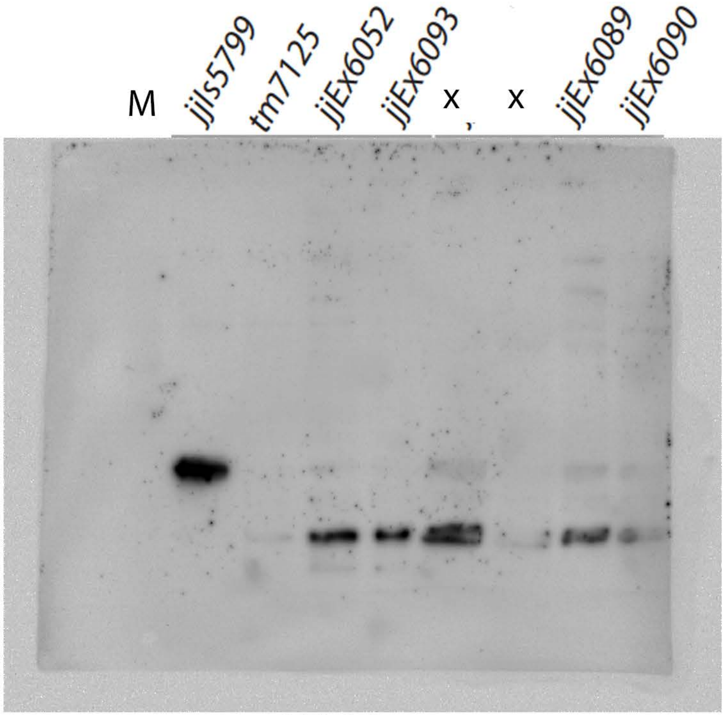

anti-FLAG

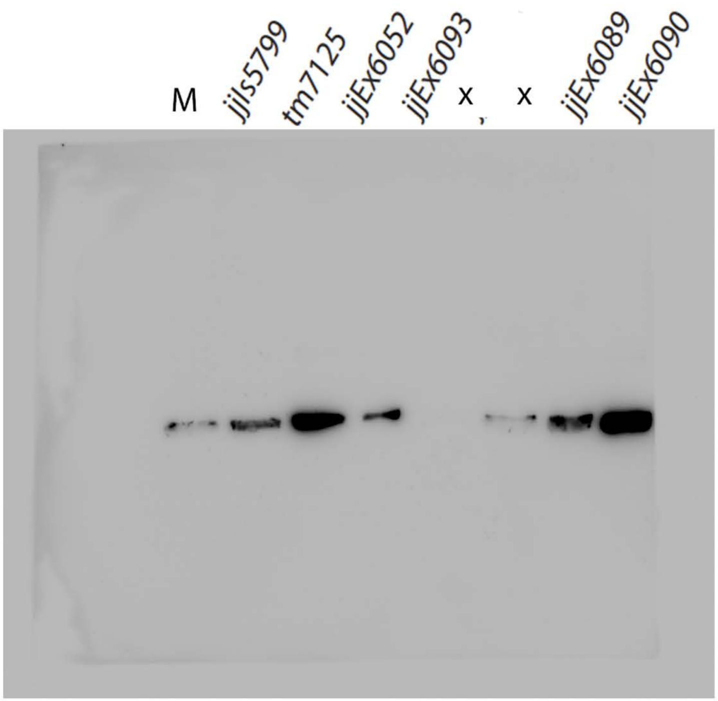

same membrane,
anti-Actin

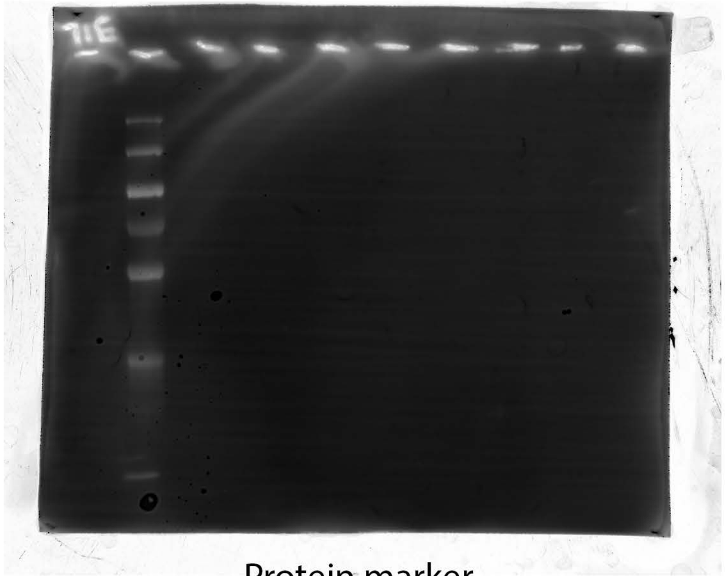

Protein marker

Raw image files for Figure 5B

	IP: Myc			IP: V5			IP: FLAG			
MW	-	+	+	-	+	+	-	+	+	V5::DBL-1pro:: FLAG::DBL-1mature
(KD)	+	-	+	+	-	+	+	-	+	HA::LON-2::Myc

IB Myc

IB V5

IB FLAG

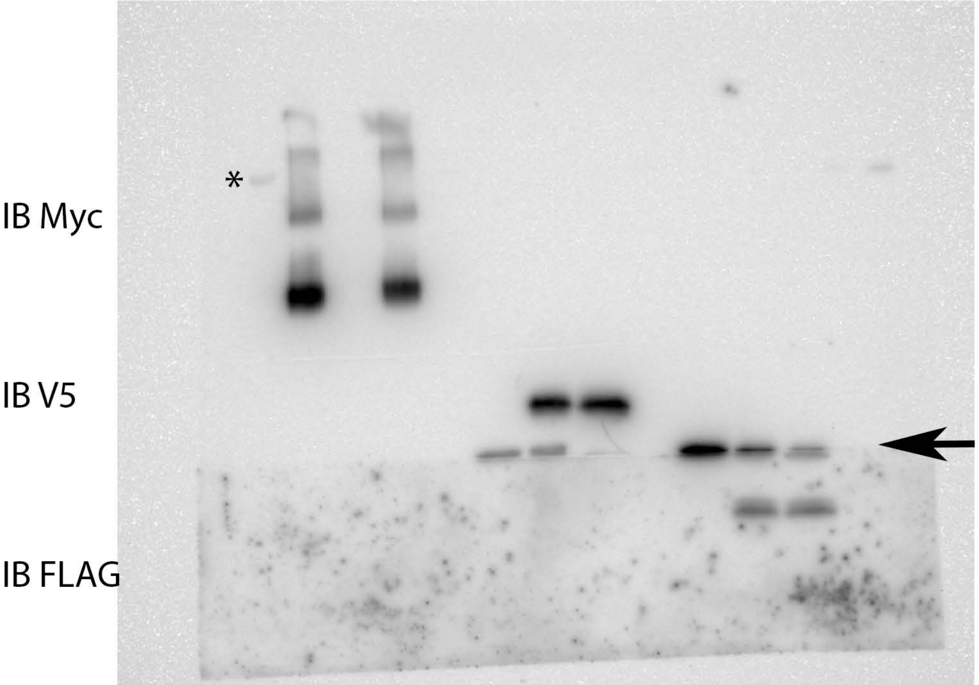

Arrow indicates mouse light chain IgG coming off beads that is detected by the secondary antibody.

* band in protein marker shows up on Western

Protein Size Marker

kD 250 130 100 70 55 35 25 15

A B C

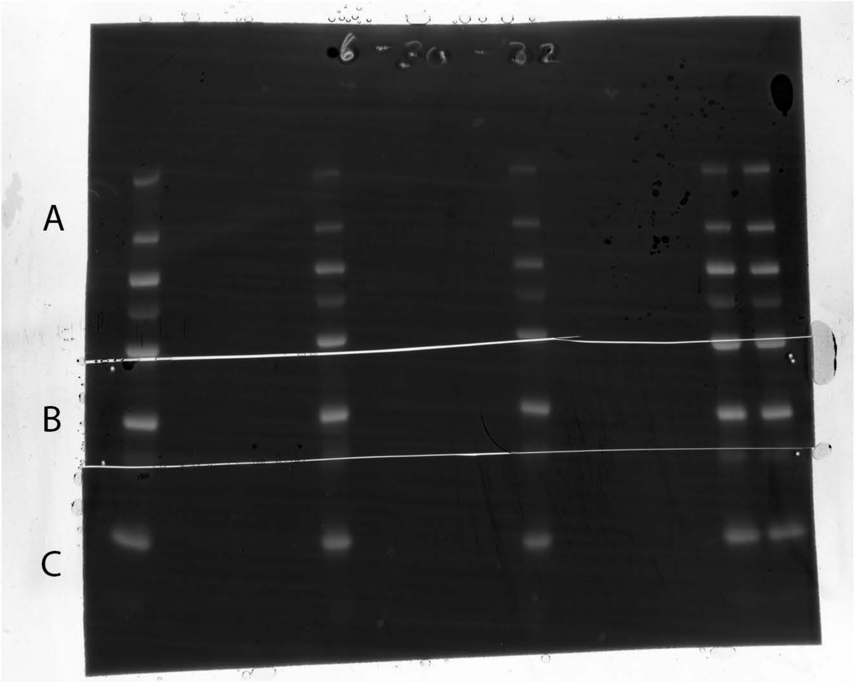

Membrane was cut into three pieces (A, B, C as marked) and probed with the three antibodies listed on the left panel.

Raw image files for Figure 5D

A

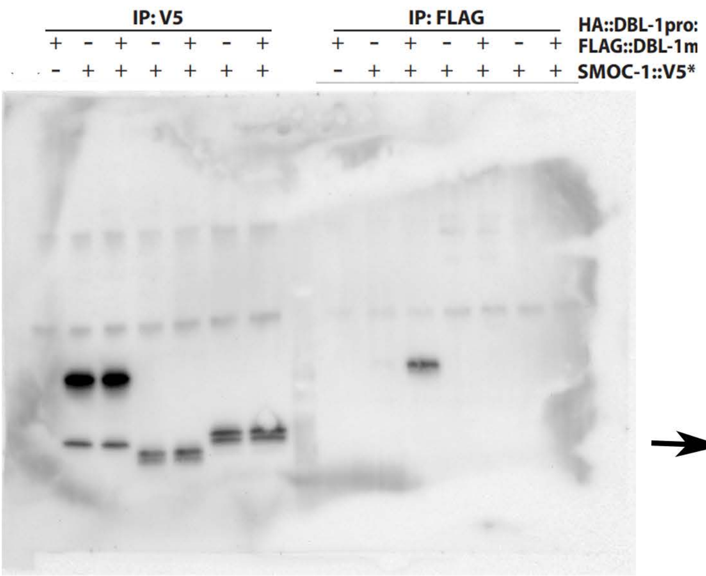

IB: anti-V5

B

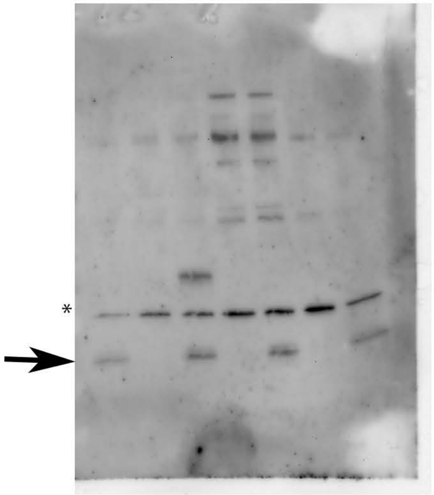

Right half of gel A was re-probed with mouse anti-FLAG antibody
* = IgG light chain
arrow = DBL-1 (mature)

C

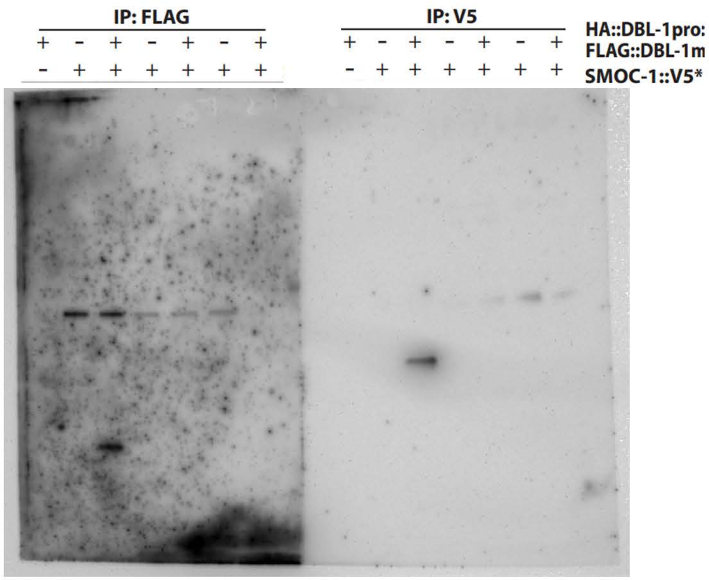

Leftover sample from Gel A (far left) were used to make a new Western blot, cut into left and right halves to probe with anti-FLAG and anti-V5.

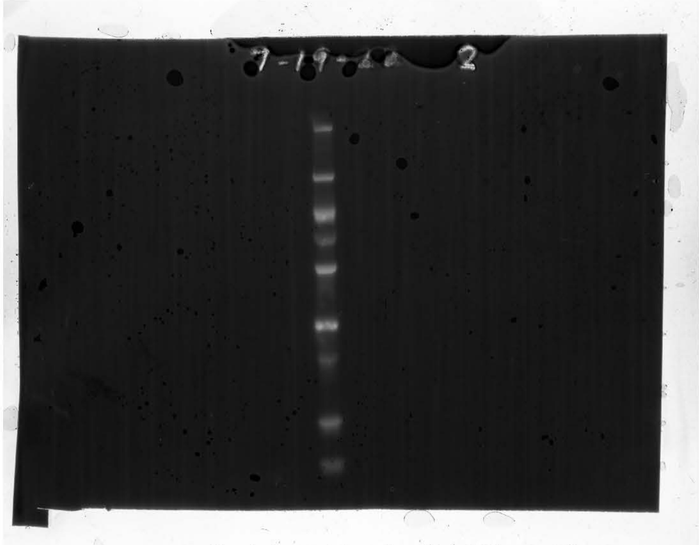

Protein marker for Gel A/B

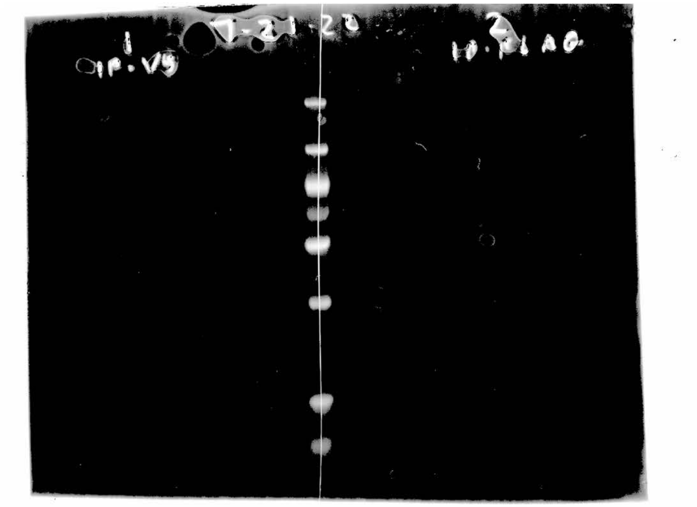

Protein Marker for Gel C

Raw image files for Figure 8D

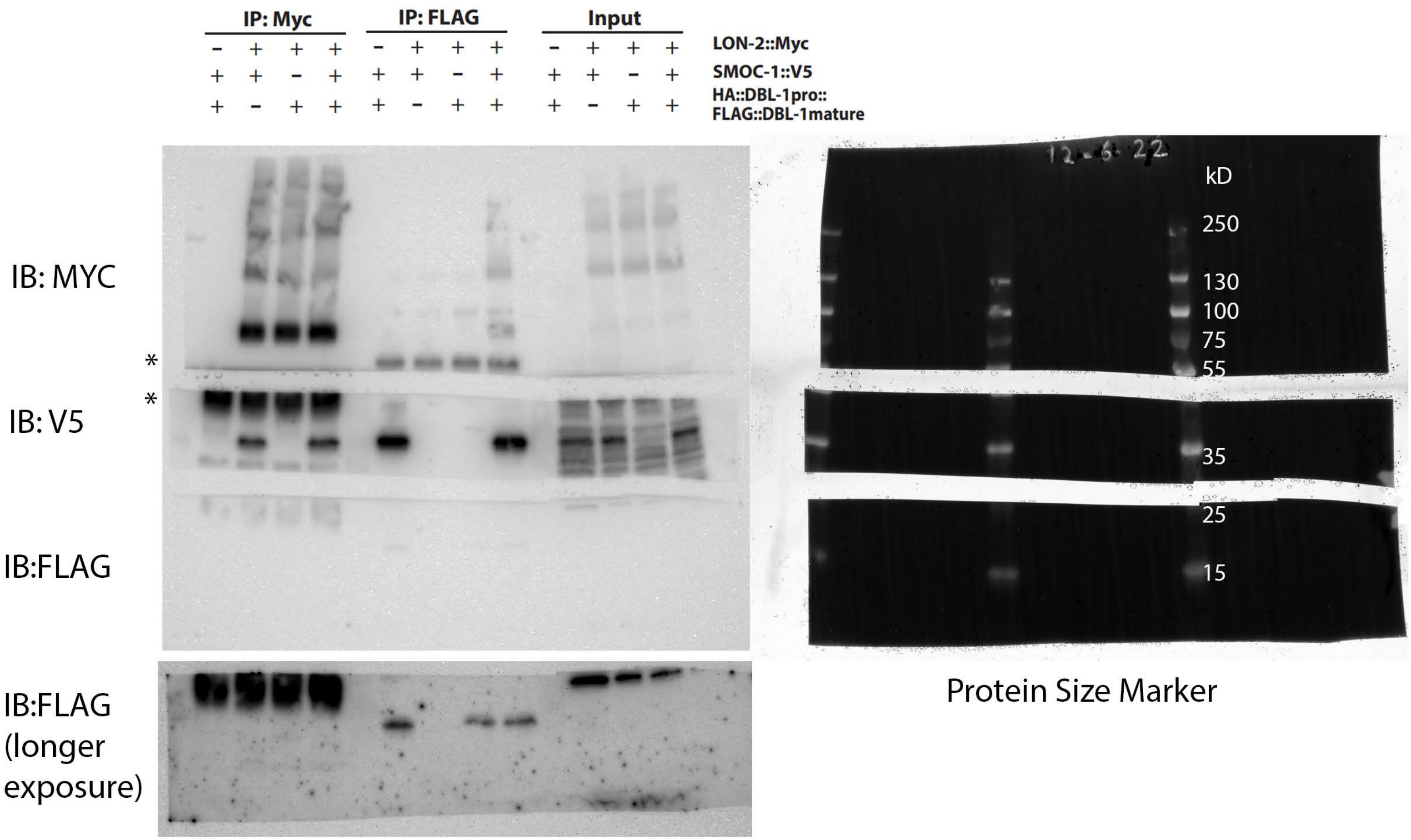

Gel cut into three sections and probed with antibodies as indicated.

* IgG heavy chain

Raw Image Files for Figure 9B

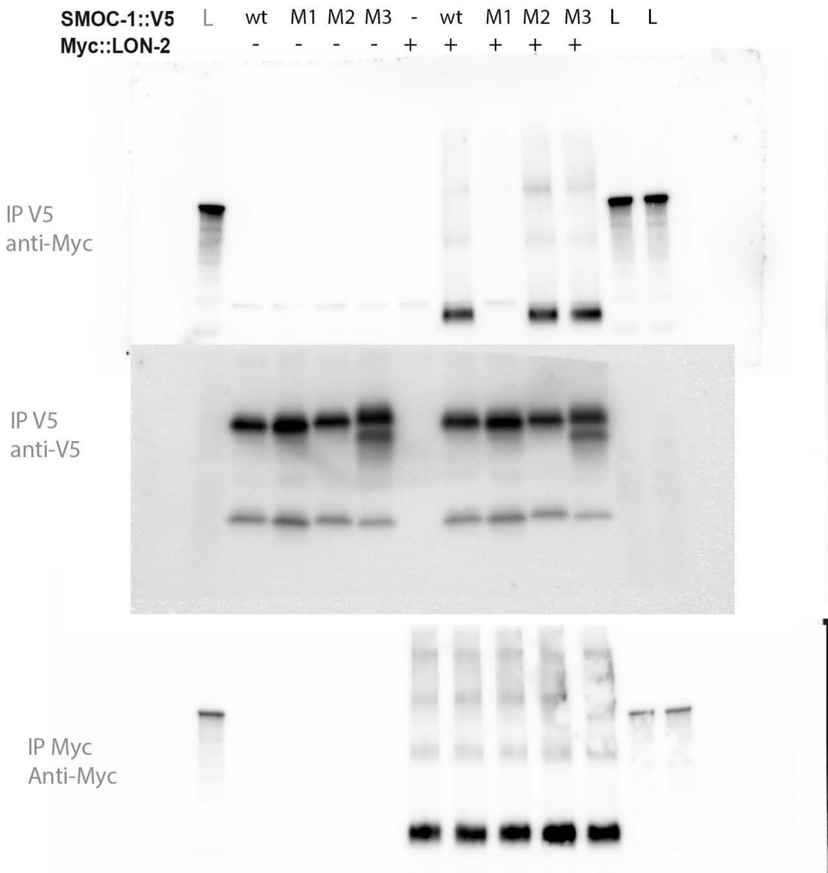

Protein Ladders

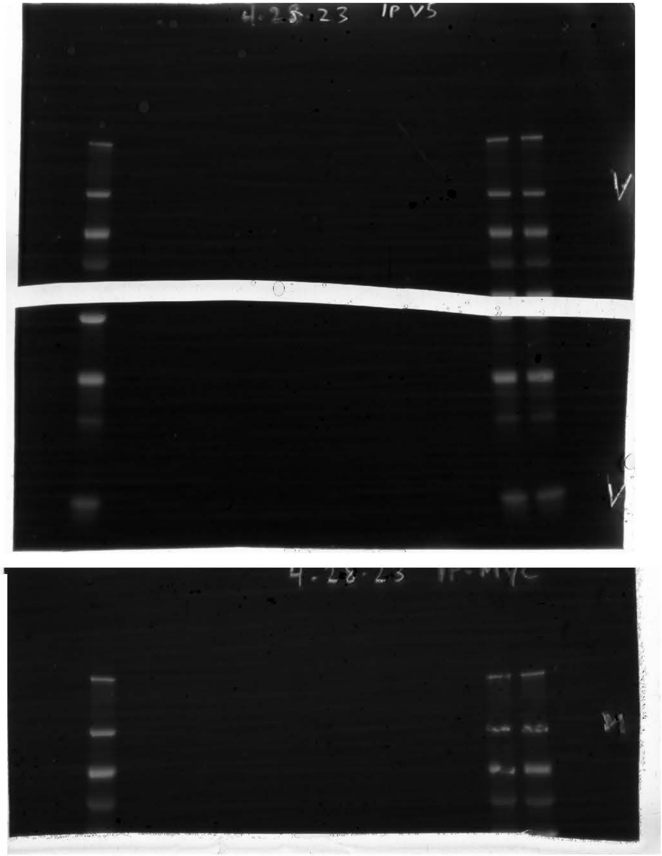

L= protein ladder lanes (there is a cross-reacting band)

Membranes were cut into two sections, top section probed with anti-Myc to detect Lon-2 and the bottom section probed with anti-V5 to detect SMOC-1.

Raw Image Files for Figure 9C

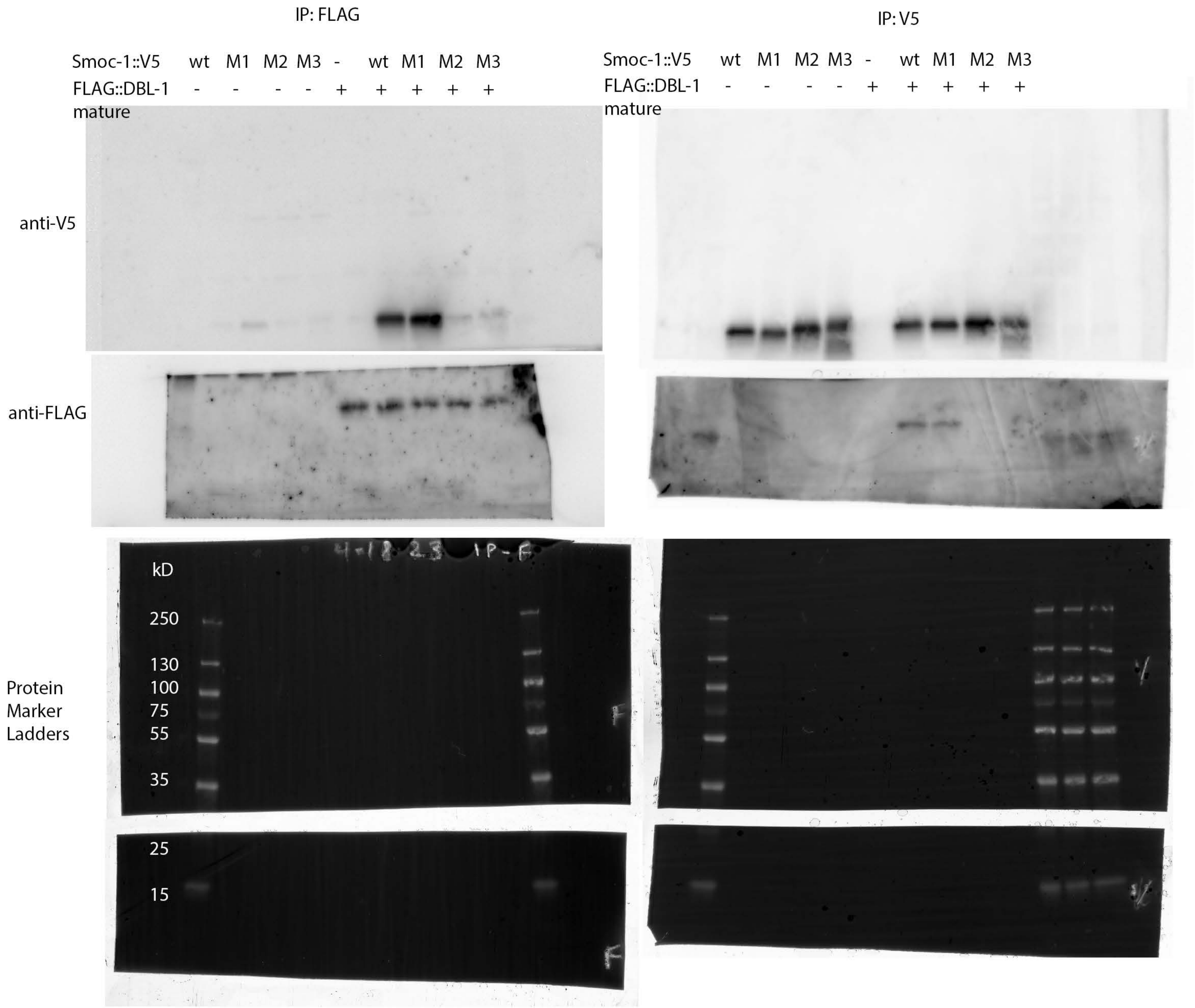

Figure 10 raw blot images

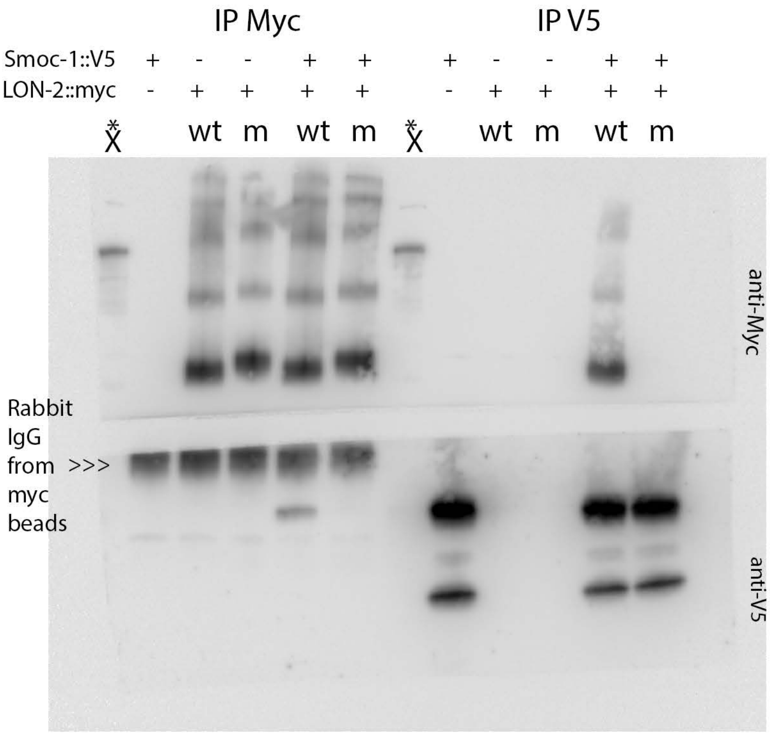

* = protein ladder (cross reaction of one band with antibody)

ECL

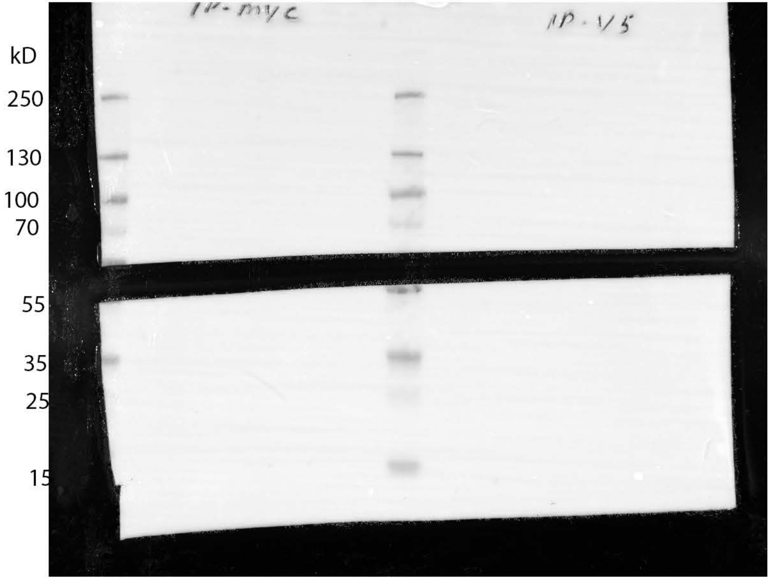

Colorimetric
(Protein ladder)

Raw Image File for Figure S2

	IP: Myc						IP: V5						
V5::DBL-1pro::	x	x	x							x	x	x	x
FLAG::DBL-1mature	-	+	+	-	+	+	-	+	+	-	+	+	
LON-2::MYC	+	-	+	+	-	+	+	-	+	+	-	+	

Anti-Myc

Anti-V5
(didn't work)

bottom half
re-probed with
anti-FLAG

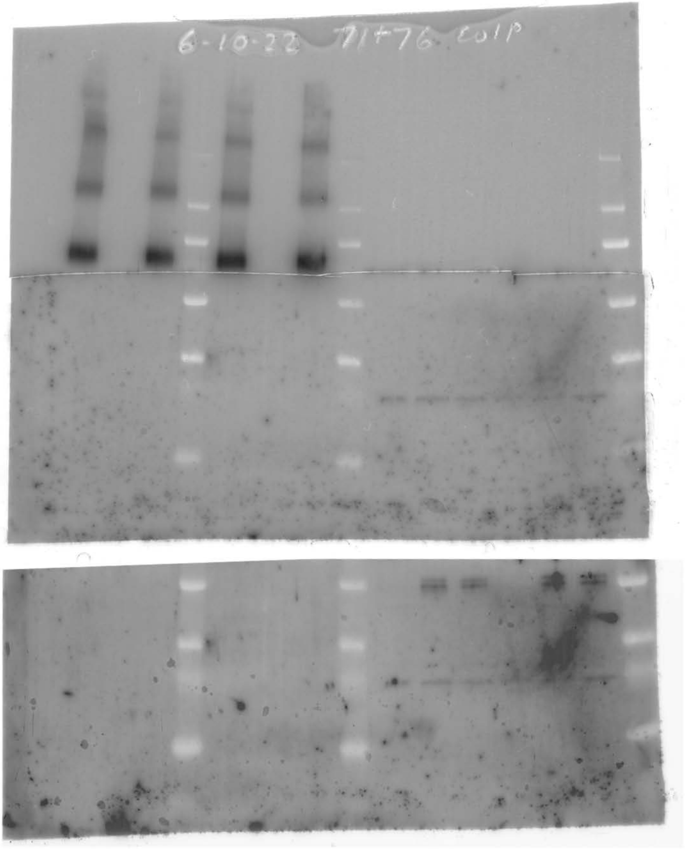

Supplement: S1 Raw Images — (PDF) [file pbio.3002272.s016.pdf]
