# Supplementary material for: SMOC-1 interacts with both BMP and glypican to regulate BMP signaling in C. elegans
Source: PLoS Biol. 2023 Aug 17;21(8):e3002272. doi: 10.1371/journal.pbio.3002272 (PMC10464977; doi:10.1371/journal.pbio.3002272)
Supplement: Attachment — Submitted filename: DeGroot et al-response letter.pdf [file pbio.3002272.s017.pdf]

Cornell University
Molecular Biology & Genetics
439/441 Biotechnology Building
Ithaca, NY 14853, USA
www.mbg.cornell.edu

Dr. Jun (Kelly) Liu
Professor
Phone: (607) 254-8942 (Office)/254-1237 (Lab)
Fax: (607) 255-6249/255-2428
E-mail: JL53@CORNELL.EDU

May 2, 2023

Dear Editor,

Enclosed please find our revised manuscript entitled "***C. elegans* SMOC-1 interacts with both BMP and glypican to regulate BMP signaling**". We very much appreciate the careful reviews from the two reviewers on our manuscript. We have added substantial amount of new data that we believe significantly strengthen our conclusions, in particular, to provide 1) both *in vivo* and *in vitro* evidence demonstrating the functional relevance of SMOC-1-LON-2 and SMOC-1-DBL-1 interactions, and 2) conservation of SMOC-BMP interactions across species, which we believe to be new and significant. We also addressed additional concerns raised by the reviewers, and revised our manuscript based on their comments and suggestions (detailed below). We hope that you will find the revised version appropriate for acceptance and publication.

Thank you very much for your time and consideration.

Sincerely yours,

Jun (Kelly) Liu
Molecular Biology and Genetics
Cornell University

Reviewer 2:

Major Criticisms:

1. While the finding reported regarding the tripartite interaction of SMOC1 with both glypicans and BMPs is potentially quite interesting, the results are based on predictive structural models, Co-IP experiments and inferences drawn from body size measurements following misexpression studies. Since this finding is novel and has not been reported in other model organisms, in vivo data to demonstrate the physical interaction between SMOC1/LON-2, and DBL-1 in developing embryos is needed to strengthen the study. When/where do the proposed interactions between SMOC1, LON-2 and DBL-1 occur in developing embryos?

We appreciate that the reviewer recognized the novelty and significance of our findings, and completely agree with the reviewer that we needed in vivo data to strengthen our results. Unfortunately, we cannot perform the recommended co-IP experiments under physiological conditions, because 1) we can only detect functionally tagged SMOC-1 and DBL-1 proteins when we overexpress them in *C. elegans*, and 2) we have not been able to generate an endogenously tagged LON-2 that is fully functional. However, we have carried out the following experiments, which we believe that, in combination, provide strong evidence indicating that SMOC-1 interacts with LON-2 and DBL-1 *in vivo*, and that the respective interactions are critical for proper functions of SMOC-1 to both positively and negatively regulate BMP signaling *in vivo*.

a) We generated site-specific mutations in SMOC-1 [SMOC-1(M1) in manuscript] that specifically disrupted its interaction with LON-2 *in vitro* (Figure 9 of the revised manuscript).
b) When we introduced the same site-specific mutations into the endogenous *smoc-1* locus, *smoc-1(M1)* mutant worms exhibit a long body size phenotype (Figure 9 of the revised manuscript).
c) We also generated site-specific mutations in LON-2 [LON-2(mut) in manuscript] that specifically disrupted its interaction with SMOC-1 *in vitro* (Figure 10 of the revised manuscript).
d) When we introduced the same site-specific mutations into the endogenous *lon-2* locus, *lon-2(mut)* mutant worms exhibit a long body size phenotype (Figure 10 of the revised manuscript).
e) Results described in (a-d) provide strong evidence that (1) the interaction between LON-2 and SMOC-1 inhibits SMOC-1 function, and (2) the major function of LON-2 in BMP signaling is to tether SMOC-1 and prevent it from positively promoting BMP signaling.
f) We also generated site-specific mutations in SMOC-1 [SMOC-1(M2) in manuscript] that specifically and significantly weakens its interaction with DBL-1 *in vitro* (Figure 9 of the revised manuscript).
g) When we introduced the M2 mutation into the endogenous *smoc-1(M1)* mutant background, *smoc-1(M1+M2)* mutant worms no longer display a long body size (Figure 9 of the revised manuscript).
h) Results described in (f-g) provide strong evidence that the positive role of BMP signaling is via SMOC-1 binding to DBL-1.

We believe that there is spatiotemporal specificity regarding the interactions between SMOC-1 and LON-2, and between SMOC-1 and DBL-1. However, as described in our manuscript, we have not been able to successfully generate fluorescent protein-tagged versions of any of the three proteins at their endogenous loci without compromising their function. For the small epitope-tagged versions of SMOC-1 and DBL-1, we could not detect the respective proteins unless we overexpress them in worms. We are actively researching other ways that would allow us to address the spatiotemporal specificity of the molecular interactions and their functional significance. We hope to report our findings in the future.

2. As the authors intent is to generalize the interaction of SMOC1 with BMPs across species, additional data is required. Data is only presented on the predicted interaction between the mature domain of DBL-1 and C. elegans SMOC-1. Using an interaction model approach, similar to that described for cSMOC1/DBL-1, it should be possible to obtain predictive interaction data for mammalian SMOC1 and BMP 2/4. Determining whether the SMOC1/BMP interaction may exist across species, or whether it is specific to the domain arrangement of C. elegans SMOC1, is significant.

We performed *in silico* structural modeling for interactions between SMOC and BMP proteins from Drosophila, Xenopus and human. High confidence interactions were predicted for SMOC-BMP (mature domain) complexes in all above species, suggesting that SMOC-BMP interaction is evolutionarily conserved in both invertebrates and vertebrates (Figure 7 of the revised manuscript). We have updated the Results section in the revised manuscript.

Minor Comments:

Line 23 - As C. elegans, Drosophila, and mammalian SMOC1 proteins have different domain organizations, to avoid confusion, please refer to C. elegans SMOC1 as cSMOC1 or C-SMOC1 throughout the manuscript.

We now refer *C. elegans* SMOC-1 in the introduction and discussion sections as CeSMOC-1 to avoid confusions. Since the Result section (except for the newly added structural modeling section) is only focused on *C. elegans* SMOC-1. We have decided to keep the original SMOC-1 name.

Line 23/24 - As the C. elegans nomenclature for the glypican, LON-2, and the BMP, DBL-1, may not be familiar to the readership, please define LON-2 and DBL-1 and their homology to mammalian glypicans/BMPs.

We have made the recommended changes, describing LON-2 as the GPC3 homolog, and DBL-1 as the BMP2/4 homolog.

Line 32 - As no data is presented to show that mammalian SMOCs can bind to BMPs, please delete the sentence "Our work provides a mechanistic basis for how the evolutionarily conserved SMOC proteins regulate BMP signaling'"

As described above, we performed *in silico* structural modeling for interactions between SMOC and BMP proteins from Drosophila, Xenopus and human. High confidence interactions were predicted for SMOC-BMP (mature domain) complexes in all above species, suggesting that SMOC-BMP interaction is evolutionarily conserved in both invertebrates and vertebrates (Results section and Figure 7 of the revised manuscript). We are therefore keeping this statement in the revised manuscript.

Line 38 - To be more concise I suggest rewording as follows; "BMP signaling is activated upon binding of secreted BMP ligands to complexes of the type I and type II BMP serine/threonine receptor kinases, which results in the intracellular phosphorylation of receptor-activated R-Smads".

We have made the recommended changes.

Line 84 -Please define the zinc finger transcription factor SMA-9 and how it is relevant to BMP signaling.

We have added additional information about SMA-9 in the introduction, pointing out that it's a Schnurri homolog and its unique relationship with the rest of the BMP core pathway in regulating body size and mesoderm (coelomocyte) development.

Line 91 - As noted earlier, as the domain structure of C. elegans SMOC1 is different to mammalian SMOC1, I suggest rewording the sentence to "C. elegans has a single SMOC protein, cSMOC-1, which is known to regulate BMP signaling'".

We have made the recommended changes.

Line 108 - I suggest changing the title to Generation of a C. elegans strain with a functionally-tagged SMOC-1.

We have made the recommended changes.

Line 191 - typo - change to …. SMOCs function by competing with BMP ligands for binding to HSPGs.

We have made the recommended changes.

Reviewer 1:

My overall take is that while the work is of very good quality, the authors didn't really nail down how SMOC proteins act as both positive and negative regulator of BMP signaling, a feature of SMOC proteins that was already known to some extent. This is based in part on the provided data relating to body size (and in some cases coelomocyte specification) but also to an absence of data that might directly bolster the hypothesized model. The IP pulldowns are clean, but it is a "non-physiological" heterologous assay. Still, as stated above, the study is well done, and the data might represent a sufficient advance for publishing in PLoS Biology; I would defer to the editors and those more immediately connected to the field of BMP signaling to make that call.

As described above in response to Reviewer 2's comments, we have carried out additional *in vitro* and *in vivo* experiments, which we believe that, in combination, provide strong evidence indicating that SMOC-1 interacts with LON-2 and DBL-1 *in vivo*, and that the respective interactions are critical for proper functions of SMOC-1 to both positively and negatively regulate BMP signaling *in vivo*.

<u>*Specific comments:*</u>

1) Statistics and presentation. In several of the figure panels the authors use symbols instead of standard asterisks to indicate significant differences. They also state in the legend that the difference between genotypes with different symbols is p<0.001 (or P<0.0001). It may be easier for most readers to rapidly comprehend the data using asterisks and bars to indicate which comparisons are being made. In fact, Figure 3C and 4B were done this way. Also, looking at the data points (e.g., Figure 1) it is somewhat hard to imagine that jjIs5119 (with a cross) vs jjIs5798 (with a dollar sign) is different by p<0.001. Ditto tm7125 vs jj269 or jj271 in Figure S1. Furthermore, in several of the figure panels and legends (e.g., Fig1 and S1) it is stated that a t-test was used to derive p values for comparisons of phenotypic percentages. t-tests are used to compare means, so this seems incorrect.

We agree with the reviewer that "*it is much easier for most readers to rapidly comprehend the [statistical] data using asterisks and bars to indicate which comparisons are being made. In fact, Figures 3C and 4B were done this way*." We used this simple presentation for the newly added Figure 10D too. However, for Figures 1C, the newly added Figure 9E-F and Figure 11B (Figure 8B in the previous version), body sizes of 6-8 different genotypes were compared, and as described in materials and methods (Lines 865-867), "an ANOVA and Tukey's honest significant difference (HSD) was used to test for differences in body size between genotypes using R (https://www.R-project.org/)". We worry that if we were to use asterisks and bars on the figures, there would be too many of them and that would cause much more confusion to the readers. We therefore chose to use different symbols to represent different homogenous groups after the test.

Using the above statistical test, the *P* value between *jjIs5798* and *jjIs5199* (shown in Figure 1) is 0.0003150 (<0.001), which was correctly stated in the figure legend. The *P* value between *tm7125*

and *jj269* (Figure S1) is 0.0000754 (<0.001) (also correctly stated), and the *P* value between *tm7125* and *jj271* is 0.0099116 (<0.01). We apologize for mis-stating the *P* value between *tm7125* and *jj271* as <0.001. We have updated the figure legend for Figure S1 to reflect this change. We have made sure to state any exceptions regarding the *P*-values in all figure legends with body size data analysis in the revised manuscript. We have also included below a screenshot of the results of statistical analysis for data shown in Figure S1 as an example of all our statistical analysis results.

	diff	lwr	upr	p adj
jj271-jj269	0.009871894	-0.008761920	0.02850571	0.5896904
N2-jj269	0.095253483	0.075846957	0.11466001	0.0000000
ok3749-jj269	-0.119168699	-0.138162272	-0.10017512	0.0000000
tm7125-jj269	0.034542810	0.013882985	0.05520263	0.0000754
N2-jj271	0.085381588	0.066085822	0.10467735	0.0000000
ok3749-jj271	-0.129040593	-0.147920985	-0.11016020	0.0000000
tm7125-jj271	0.024670915	0.004115096	0.04522673	0.0099116
ok3749-N2	-0.214422181	-0.234065587	-0.19477878	0.0000000
tm7125-N2	-0.060710673	-0.081969457	-0.03945189	0.0000000
tm7125-ok3749	0.153711508	0.132829016	0.17459400	0.0000000

As described in materials and methods (Lines 873-876), when we scored the penetrance of the Susm phenotypes, we scored “three to seven plates of worms….”, and then calculated the means for each genotype, and used *t*-tests to compare the means. We are sorry that we did not make this clear. We have since added in the revised manuscript the following phrase in Line 876 “…*and the means of the Susm penetrance were* [presented]”.

2) Although I understand why the authors use the term "Susm penetrance" and "rescue of Susm" as a read out for SMOC-1-BMP activity, I wonder if it would be simpler for most readers to provide either the average number of posterior (M-derived) coelomocytes or to state the percentage of worms with 1-2 posterior coelomocytes. I think it can be difficult for readers who aren't geneticists to do the mental gymnastics required interpret the findings and the above presentation method may be more straightforward.

We appreciate the reviewer’s point of view. As described in our answers to point 3) below, the Susm phenotype is not straightforward to understand and we still do not have a full grasp of the underlying molecular mechanism. We have tried in our previous presentations by focusing on the number of CCs in different mutant animals. That way of presentation actually caused more confusion among audience who are not *C. elegans* geneticists. Because mutations in BMP pathway components exhibit a body size phenotype, and can specifically suppress the *sma-9(0)* CC phenotype (Susm), we think that by referring to the Susm as a phenotype and the degree of Susm as a penetrance of the phenotype, it is much simpler to convey whether or not a mutation disrupts the function of BMP signaling.

3) The apparent positive and negative roles of SMOC-1 in BMP signaling could be laid out more clearly in the introduction and throughout. It will likely be confusing to many readers as to whether SMOC-1 acts as a positive or negative regulator of BMP signaling in the coelomocyte assay, which is used but not well discussed. Some commentary about differences between these BMP pathways is warranted given that several of the pathway components, including lon-2 and sma-9, do not act in

the way that one would expect in the M lineage and this gets quite confusing. For example, why does loss of lon-2 (along with smoc-1 and dbl-1) suppress the sma-9 defect? In other words, please summarize and potentially diagram what is known from previous studies to help readers frame the results. Moreover, how do the current findings apply to a model for the coelomocyte assay?

We understand the confusion of the reviewer regarding the role of BMP signaling in regulating the coelomocyte (CC) fate specification. In fact, we have been working to address this question since we first discovered this phenotype almost 20 years ago. Unfortunately, we still do not understand the exact mechanism. What we do know are the following (based on our multiple prior publications: Foehr et al., 2006; Foehr and Liu, 2008; Tian et al., 2010; Liu et al., 2015; Wang et al., 2017; Shen et al., 2018; DeGroot et al., 2019; Liu et al., 2020, 2022):

- SMA-9 is a homolog of Schnurri, which in Drosophila and vertebrates function as a co-factor of Smads.
- *sma-9(0)* mutations cause a small body size phenotype, similar to phenotypes in mutants with decreased BMP signaling, but none of the other mutations in any BMP pathway components, except for *sma-9(0)*, exhibit a CC phenotype.
- Mutations in all BMP pathway components, whether cause a decrease of BMP signaling or increase of BMP signaling based on the body size phenotype, can all suppress the *sma-9(0)* CC phenotype, i.e, exhibit a Susm phenotype.
- For the same mutation in a given gene in the BMP pathway, the penetrance of suppression does not correlate with the body size. For example, *smoc-1(0)* mutants have a ~5% decrease of body size, but have a ~98% penetrance of the Susm phenotype, while *lon-2(0)* mutants have a ~15% increase in body size, but have a ~90% penetrance of the Susm phenotype.
- Despite these paradoxical observations, the Susm phenotype is highly *sensitive* and *specific* to mutations in BMP pathway components.

Since the focus of the current manuscript is on SMOC-1, we feel that getting too much into the details of SMA-9 will divert the focus of the main story. We did modify the introduction section in the revised manuscript, by expanding a bit more on SMA-9 and the Susm phenotype.

4) Overall, the protein interactions data (MS and IP) were thorough and presented well. Will the full MS dataset be included as supplementary data? What else was pulled out? Also, explaining a little more about how the double-tagged DBL-1 works would be useful. Would FLAG be bound or detected in both the mature and unprocessed forms? Is there a reason to test/show/state if SMOC-1 can bind to full length DBL-1 as was assayed for LON-2 in Fig S2?

We have added two supplemental tables (Tables S2 and S3), attaching the full MS datasets from the two experiments that performed, the first one using transgenic worms overexpressing an untagged SMOC-1 and the FLAG-tagged full length SMOC-1, while the second one transgenic worms overexpressing an untagged SMOC-1, the FLAG-tagged full length SMOC-1, and the FLAG-tagged SMOC-1(EC). We have sorted the data based on fold enrichment of proteins in the tagged vs. untagged samples.

BMP molecules such as DBL-1 are composed of a prodomain and a mature domain. Furin proteases cleave the BMP molecules when they are being secreted, releasing the prodomain and the mature domain. In the S2 cell expression system (as described in the materials and methods section), if we harvest cell lysates, we will be able to detect full length DBL-1, using either anti-HA or anti-FLAG antibodies. When we harvest cell media, which contain secreted proteins, anti-HA can only detect the prodomain, while anti-FLAG can only detect the mature domain.

We did not test for interaction between SMOC-1 and full-length DBL-1 because previously published data from us and others have shown that the two proteins are not expressed in the same cells: *smoc-1* is expressed in the pharynx, in intestinal and tail hypodermal cells (DeGroot et all, 1029), while *dbl-1* is expressed in the ventral nerve cord (Suzuki et al., 1999). Furthermore, our structural modeling did not detect any interaction between SMOC-1 with either full length or the mature domain of DBL-1. We did try co-IP between LON-2 with all versions of DBL-1 because of the previous reported LON-2 interaction with mammalian BMP2. But we failed to reproduce that result. In addition, our structural modeling did not detect any interaction between LON-2 and any forms of DBL-1.

5) In Figure 3C/D, is it necessary to show the size or Susm phenotype of siblings that didn't carry the array? To me this may add some unnecessary confusion for some readers, although I understand that it can provide a good internal control on that day. In addition, does OE of smoc-1 TY in a wild type cause a reduction in size? In other words, is there any indication of a dominant negative or antimorphic effect, particularly given some suggestion of this in Figure 4B?

We have decided to keep the data for non-transgenic sibling in Figure 3. For rigor and reproducibility, whenever we conduct experiments using non-integrated transgenic lines, we feel that it is important to score and present both the transgenic and non-transgenic siblings, using the non-transgenic worms as internal controls.

We have found that overexpression of SMOC-1(TY) in WT animals does not produce a reduction in body size, similar to what we have shown in Figure 3, when overexpressing SMOC-1(TY) in the *smoc-1(null)* background. We therefore do not believe that SMOC-1(TY) has an anti-morphic effect. At present, we do not know why expression of SMOC-1(TY) at the endogenous level resulted in a shorter body size than *smoc-1(0)* null animals (Figure 4). We plan to address this question in the future.

6) Regarding the model, if "SMOC-1 has both a negative, LON-2-dependent role and a positive, LON-2-independent role in regulating BMP signaling", then wouldn't you expect at least some additivity in the lon-2 mutant that also overexpresses SMOC-1? This scenario wasn't included in Figure 8A, but it seems that less LON-2 should mean more diffusible DBL-1. Along those lines and in part for completeness, what is the phenotype of DBL-1 OE in a lon-2 mutant background? Regarding the statistics in 8B and the Figure legend, and as discussed above, it is stated that the difference between groups with different symbols is p <0.0001. But this seems very unlikely when comparing, for example, DBL-1 OE vs DBL-1 OC + SMOC-1 OE. Although there could a statically

significant difference between these conditions, it would seem to be much more minimal based on the data point spread, and this difference is used in part to suggest a positive role for SMOC-1 in DBL-1 signaling. It also wasn't entirely clear how OE of SMOC-1(EC) stimulates BMP given that binding to DBL-1 wasn't observed in S2 system, which is presumably expressing the components at fairly high levels.

Based on our new *in vitro* and *in vivo* data described above, we have modified the model, as shown in Figure 12 of the revised manuscript, to better illustrate how SMOC-1 can both positively and negatively regulate BMP signaling. We believe that the newly added CRISPR results at the endogenous *smoc-1* and *lon-2* loci provide much clearer and unambiguous results regarding the functional significance of LON-2-SMOC-1 and DBL-1-SMOC-1 interactions. In light of our new data and given that our *smoc-1(OE)* and *dbl-1(OE)* lines may drastically overexpress the gene of interest, it is conceivable that LON-2 level might not matter anymore in the *smoc-1(OE)* and *dbl-1(OE)* overexpression strains.

We have examined the body size of *dbl-1(OE); lon-2(0)* worms and have included the results in the revised Figure 11. We have found that the body sizes of *dbl-1(OE); lon-2(0)* worms are significantly different from the body sizes of *smoc-1(OE); lon-2(0)* worms.

We apologize that we did make a mistake in the original Figure 8B. The scale of the Y axis was incorrectly set between 0.8 and 1.3 when we used R to make the ggplot. As a result, some data points with values greater than 1.3 were left out of the original graph. Figure 11 in our revised manuscript, which shows the body sizes of the same overexpression strains as well as the newly added *dbl-1(OE); lon-2(0)* strain, now with the scale of the Y axis correctly set between 0.8 and 1.5. We hope that it shows more clearly the difference between the body sizes of *dbl-1(OE)* vs. *smoc-1(OE); dbl-1(OE)* worms. The P value between them is 0.0009930.

We are also puzzled by the fact that overexpression of SMOC-1(EC) led to a long body size phenotype (Figure 3). We suspect three reasons: 1) Even though the proteins are both overexpressed in the *Drosophila* S2 system and in *C. elegans*, we cannot directly compare how much each protein is over-expressed in each system. Our *C. elegans* transgenic strains may have higher expression levels of SMOC-1(EC) than *Drosophila* S2 cells. 2) The IP condition that we used is quite stringent, which may preclude possible low-affinity interactions between SMOC-1(EC) and DBL-1 that can happen in *C. elegans*. 3) We cannot rule out any additional proteins present in *C. elegans* that may facilitate the interactions between SMOC-1(EC) and DBL-1. We suspect that the spatial and temporal regulation of SMOC-1 function is also important, which may be one of the underlying causes for the overexpression results. We hope that our continued research will help address this question. We have updated this part of the Discussion Section in the revised manuscript.

Very minor comments:

- Figure S2. Remove the word "either" from the title.

We have made the recommended changes.

- Figure 3A did not appear to be referenced in the text.

We have made the recommended changes.

- In Figure 3C/D, is it necessary to show the size or Susm phenotype of siblings that didn't carry the array? To me this may add some unnecessary confusion for some readers, although I understand that it can provide a good internal control on that day.

As described earlier, we have decided to keep the data for non-transgenic siblings in this figure. For rigor and reproducibility, whenever we conduct experiments using non-integrated transgenic lines, we feel that it is important to score both the transgenic and non-transgenic siblings, using the non-transgenic worms as internal controls.

- Line 337 change vertebrates to vertebrate.

We have made the recommended changes.

- Figure S1B. Uncapitalize smoc-1(jj276…)

We have made the recommended changes.
