# Supplementary material for: SMOC-1 interacts with both BMP and glypican to regulate BMP signaling in C. elegans
Source: PLoS Biol. 2023 Aug 17;21(8):e3002272. doi: 10.1371/journal.pbio.3002272 (PMC10464977; doi:10.1371/journal.pbio.3002272)
Supplement: Attachment — Submitted filename: DeGroot et al-response letter-7-13-2023.pdf [file pbio.3002272.s018.pdf]

Cornell University
Molecular Biology & Genetics
439/441 Biotechnology Building
Ithaca, NY 14853, USA
www.mbg.cornell.edu

Dr. Jun (Kelly) Liu
Professor
Phone: (607) 254-8942 (Office)/254-1237 (Lab)
Fax: (607) 255-6249/255-2428
E-mail: JL53@CORNELL.EDU

July 10, 2023

Dear Editor,

Enclosed please find our revised manuscript entitled "**SMOC-1 interacts with both BMP and glypican to regulate BMP signaling in *C. elegans***". We addressed the questions raised by the reviewer and revised our manuscript accordingly (detailed below). In addition, we have gone through the reference list and found none that was retracted. However, we did add two new references that we accidentally omitted in the previous submission. We hope that you will find the revised version appropriate for acceptance and publication.

Thank you very much for your time and consideration.

Sincerely yours,

Jun (Kelly) Liu
Molecular Biology and Genetics
Cornell University

From the Editorial Team:

We would like you to consider a suggestion to improve the title:
"SMOC-1 interacts with both BMP and glypican to regulate BMP signaling in C. elegans"

We have changed the title as suggested.

Reviewer 2:

<u>*Major Criticism:*</u>

Although the authors were unable to present direct, in vivo, evidence for the tripartite interaction of CeSMOC-1, LON-2 and DBL-1 in C. elegans, they provide additional indirect evidence in the revised manuscript to suggest that this is possible (Figures 9 and 10 of the revised manuscript). While the response provides additional support for the tripartite model, the authors state that none of the possible forms of DBL-1 (mature domain, pro-domain, or full-length) were predicted to interact with LON-2/glypican. However, the authors point out that previous studies have shown that Drosophila DPP and mammalian BMP2/4 do interact with glypicans/HSPGs. As the disparity is compelling, please present additional in silico structural modeling data to compare the predicted interactions between DBL-1/LON-2 and BMP2/4/LON-2. Are the sequences in DPP and BMP2/4 predicted to interact with HSPGs conserved in DBL-1?

1) We have conducted additional *in silico* structural modeling between LON-2 and DBL-1 (including full length, the prodomain and the mature domain of DBL-1), and between human LON-2 and DBL-1 homologs, and found no interaction between each pair of proteins. The results are presented in Figure S3 of the revised manuscript.

2) Previous studies have shown that *Drosophila* DPP and vertebrate BMP2/4 interact with glypicans/HSPGs. In particular, it's a stretch of positively charged amino acids at the N-terminus of the DPP or BMP mature domain that can bind to the heparin glycosaminoglycan. Any functional studies published were using either transgenes (in flies) or explants (Xenopus) that overexpressed the wildtype or mutant BMP proteins. DBL-1 also contains a stretch of basic residues in similar locations (see Figure below). However, since our CRISPR mutant in LON-2 that specifically disrupted LON-2-SMOC-1 interaction caused a LONG mutant phenotype indistinguishable from the LONG phenotype exhibited by *lon-2* null mutants, and since our LON-2 mutations do not appear to affect the glycosylation patterns of LON-2 when expressed in Drosophila S2 cells, we believe that even if DBL-1 can bind to LON-2 via the sugar modifications in LON-2, the interaction between the LON-2 protein core and SMOC-1 is primarily responsible for the inhibitory functions of LON-2 in regulating body size and BMP signaling. Future work exploring the *in vivo* functional relevance of the sugar modifications on LON-2 and of the basic residues in DBL-1 will be needed, but is beyond the scope of the current manuscript.

Boxed area is from Akiyama et al (2008) Developmental Biology 313: 408-419

Xenopus	BMP4	SPKQQRPRKKNKHC
Human	BMP4	SPKHHSQRARKKNKNC
Mouse	BMP4	SPKHHPQRSRKKNKNC
Zebrafish	BMP4	SPKQRGRKRNRNC
Drosophila	Dpp	DVSGGEGGGKGGRNKRHARRPTRRKNHDDTC
	Dpp$^{\Delta N}$	DVSGGEGGGKGGRNKRHA-------NHDDTC

Boxed area below is from Ohkawara et al (2002) Current Biology 12:205-209

**BMP-2/4 subfamily**	
Xenopus BMP-2	QARHKQRKRLKSSC
Xenopus BMP-4	SPKQQRPRKKNKHC
human BMP-4	SPKHHSQRARKKNKNC
mouse BMP-4	SPKHHPQRSRKKNKNC
chick BMP-4	SPKHHGSRKNKKNC
zebrafish BMP-4	SPKQRGRKRNRNC
fly DPP	DVSGGEGGGKGGRNKRHARRPTRRKNHDDTC
**BMP-4 variants**	
Xenopus WTBMP-4	SPKQQRPRKKNKHC
Δ1BMP-4	SP--------NKHC
Δ2BMP-4	SPKQQRP---NKHC

DBL-1 **SAQTGNSERKNRKKGRKHHNTEAESNLC**

Minor Criticism

The authors now refer to C. elegans SMOC-1 as CeSMOC-1 in the introduction and discussion, but not in the results section. To avoid confusion, please change SMOC-1 to CeSMOC-1 throughout the manuscript, when referring to results obtained with CeSMOC-1.

While we understand the reviewer's intention, there are also *C. elegans* nomenclature rules that we must follow. We have gone through the entire manuscript and used CeSMOC-1 whenever it is ambiguous as to which SMOC protein we are referring to, in particular in the Introduction and Discussion sections. We have kept the name SMOC-1 in most of the Results section when we were only discussing *C. elegans* SMOC-1.
